# Hepatic overproduction of 13-HODE due to ALOX15 upregulation contributes to alcohol-induced liver injury in mice

**DOI:** 10.1038/s41598-017-02759-0

**Published:** 2017-08-21

**Authors:** Wenliang Zhang, Wei Zhong, Qian Sun, Xinguo Sun, Zhanxiang Zhou

**Affiliations:** 1 Center for Translational Biomedical Research, University of North Carolina Greensboro, North Carolina Research Campus, Kannapolis, NC 28081 USA; 2 Department of Nutrition, University of North Carolina at Greensboro, North Carolina Research Campus, Kannapolis, NC 28081 USA

## Abstract

Chronic alcohol feeding causes lipid accumulation and apoptosis in the liver. This study investigated the role of bioactive lipid metabolites in alcohol-induced liver damage and tested the potential of targeting arachidonate 15-lipoxygenase (ALOX15) in treating alcoholic liver disease (ALD). Results showed that chronic alcohol exposure induced hepatocyte apoptosis in association with increased hepatic 13-HODE. Exposure of 13-HODE to Hepa-1c1c7 cells induced oxidative stress, ER stress and apoptosis. 13-HODE also perturbed proteins related to lipid metabolism. HODE-generating ALOX15 was up-regulated by chronic alcohol exposure. Linoleic acid, but not ethanol or acetaldehyde, induced ALOX15 expression in Hepa-1c1c7 cells. ALOX15 knockout prevented alcohol-induced liver damage via attenuation of oxidative stress, ER stress, lipid metabolic disorder, and cell death signaling. ALOX15 inhibitor (PD146176) treatment also significantly alleviated alcohol-induced oxidative stress, lipid accumulation and liver damage. These results demonstrated that activation of ALOX15/13-HODE circuit critically mediates the pathogenesis of ALD. This study suggests that ALOX15 is a potential molecular target for treatment of ALD.

## Introduction

Excessive alcohol consumption has long been associated with the development of liver diseases, including steatosis (fatty liver), hepatitis, fibrosis, cirrhosis, and hepatocellular carcinoma^1–3^. Fatty liver as the first stage of alcoholic liver disease (ALD) may not cause any symptoms, but generation of toxic lipid species at this stage plays critical role in the initiation and progression of ALD^4^.

Chronic alcohol exposure disrupts lipid homeostasis in the liver through multiple lipid metabolic pathways, including *de novo* lipogenesis, fatty acid oxidation, lipid uptake, very low-density lipoprotein export, and detoxification^5–8^. Alcohol exposure also impairs lipid storage function of white adipose tissue (WAT), leading to lipodystrophy^8, 9^. Our previous study showed that triglycerides stored in WAT could be released and deposited in the liver upon alcohol exposure^9^. It has been suggested that accumulation of excessive lipids in the liver plays an important role in the initiation and progression of ALD.

Hepatocyte apoptosis has been well documented in both alcoholic patients and animal models of ALD^10–12^. Mechanistic studies showed that hepatic apoptosis could be triggered by a variety of signal pathways, including endoplasmic reticulum (ER) stress, cell death receptor-mediated signaling cascade, reactive oxygen species (ROS) generation, lysosomal permeabilization, and mitochondrial dysfunction^12–16^.

Alcohol consumption induces oxidative stress and increases free fatty acid levels with linoleic acid being mostly elevated in the liver^5^. Peroxidation of fatty acids via arachidonate 15-lipoxygenase (ALOX15) generates bioactive lipid derivatives such as 13-hydroxyoctadecadienoic acid (13-HODE) from linoleic acid, and 12-hydroxyeicosatetraenoic acids (12-HETE) from arachidonic acid^17^. Disruption of ALOX15 has been shown to protect mice from nonalcoholic fatty liver disease and high fat diet-induced steatohepatitis^18, 19^. A human study reported that plasma levels of 9- and 13-HODE are elevated in patients with ALD^20^.

Although it has been shown that chronic alcohol feeding induces both lipid dyshomeostasis and hepatic apoptosis, the precise molecular mechanisms linking alcohol-induced lipid dyshomeostasis and hepatic apoptosis have not been fully defined.

The present study was designed to define the role of ALOX15/13-HODE in the pathogenesis of ALD and to test the potential of ALOX15 as a molecular target in the treatment of ALD.

## Results

### Increased hepatic HODE levels are associated with alcohol-induced liver injury in mice

**Figure 1:**
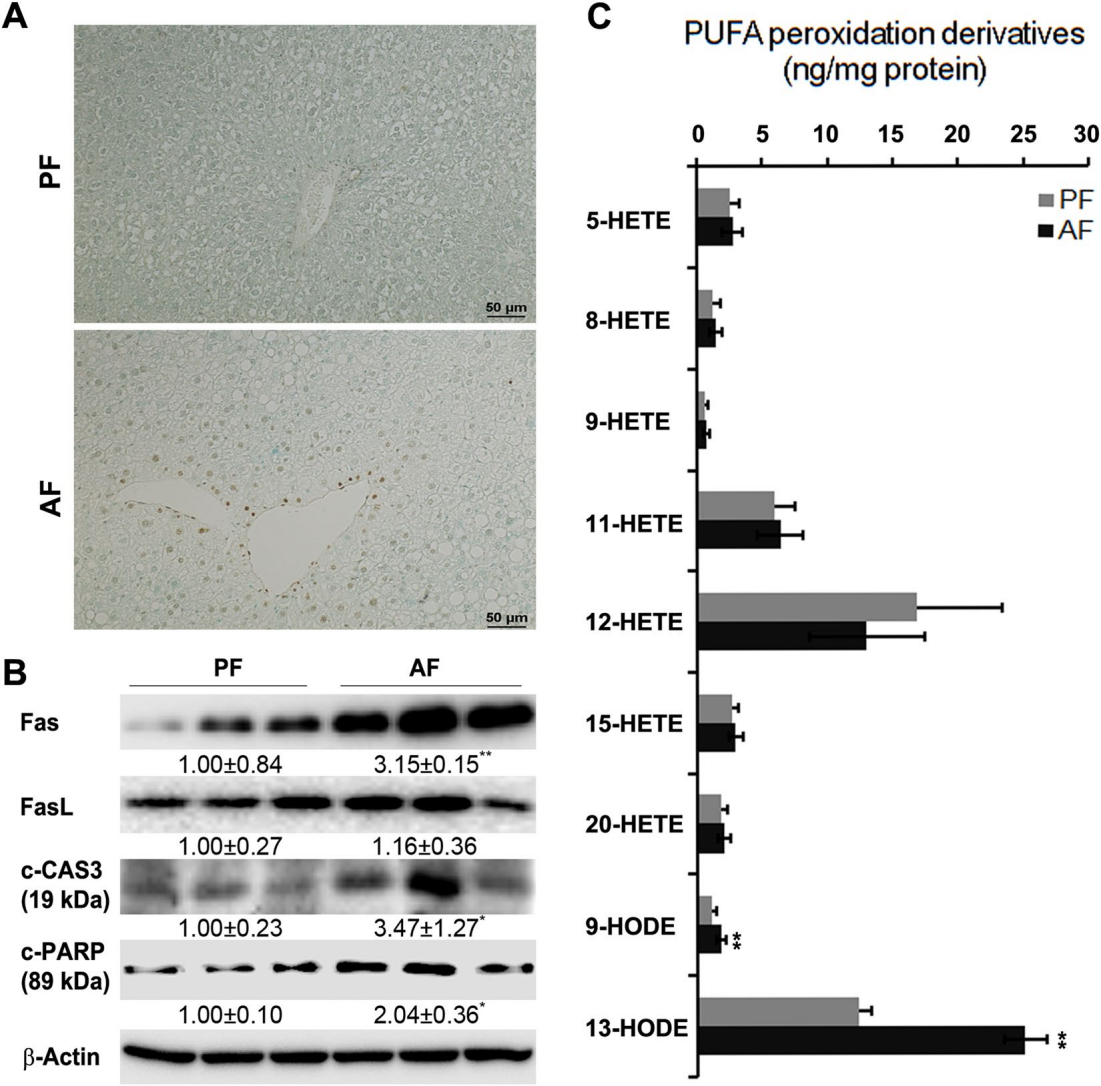
Chronic ethanol feeding induced hepatic apoptosis and increased hepatic HODE levels. WT male mice were fed with control (PF) or alcohol (AF) liquid diet for 8 weeks. (**A**) TUNEL staining of liver sections. (**B**) Immunoblot of proteins involved in apoptosis. Protein levels were quantitated by NIH image J. (**C**) Hepatic HODE and HETE levels measured by LC-MS. All values are denoted as means ± SD (n = 3 in B, n = 6 in C). *P < 0.05, **P < 0.01. HETE: hydroxyeicosatetraenoic acids, HODE: hydroxyoctadecadienoic acid, PUFA: polyunsaturated fatty acids.

Chronic alcohol feeding induced liver damage as indicated by elevated plasma ALT (PF: 10.9 ± 1.4 U/L vs. AF: 69.4 ± 15.8 U/L) and AST (PF: 12.9 ± 2.7 U/L vs. AF: 46.4 ± 9.4 U/L) levels. TUNEL assay of apoptotic cell death showed strong positive staining in the liver of AF mice but not in the PF mice (Fig. 1A). Immunoblot analysis showed that chronic ethanol feeding significantly increased protein levels of Fas, c-CAS3, and c-PARP in AF mice compared with that of PF mice (Fig. 1B). Alcohol feeding significantly increased the levels of both 9-HODE (1.11 ± 0.44 vs. 1.87 ± 0.33 ng/mg protein) and 13-HODE (12.38 ± 0.95 vs. 25.05 ± 1.67 ng/mg protein), but did not affect all the seven HETE species detected in the liver (Fig. 1C).

### 13-HODE increases ROS, ER stress, and apoptosis, and alters lipid metabolism in Hepa-1c1c7 cells

**Figure 2:**
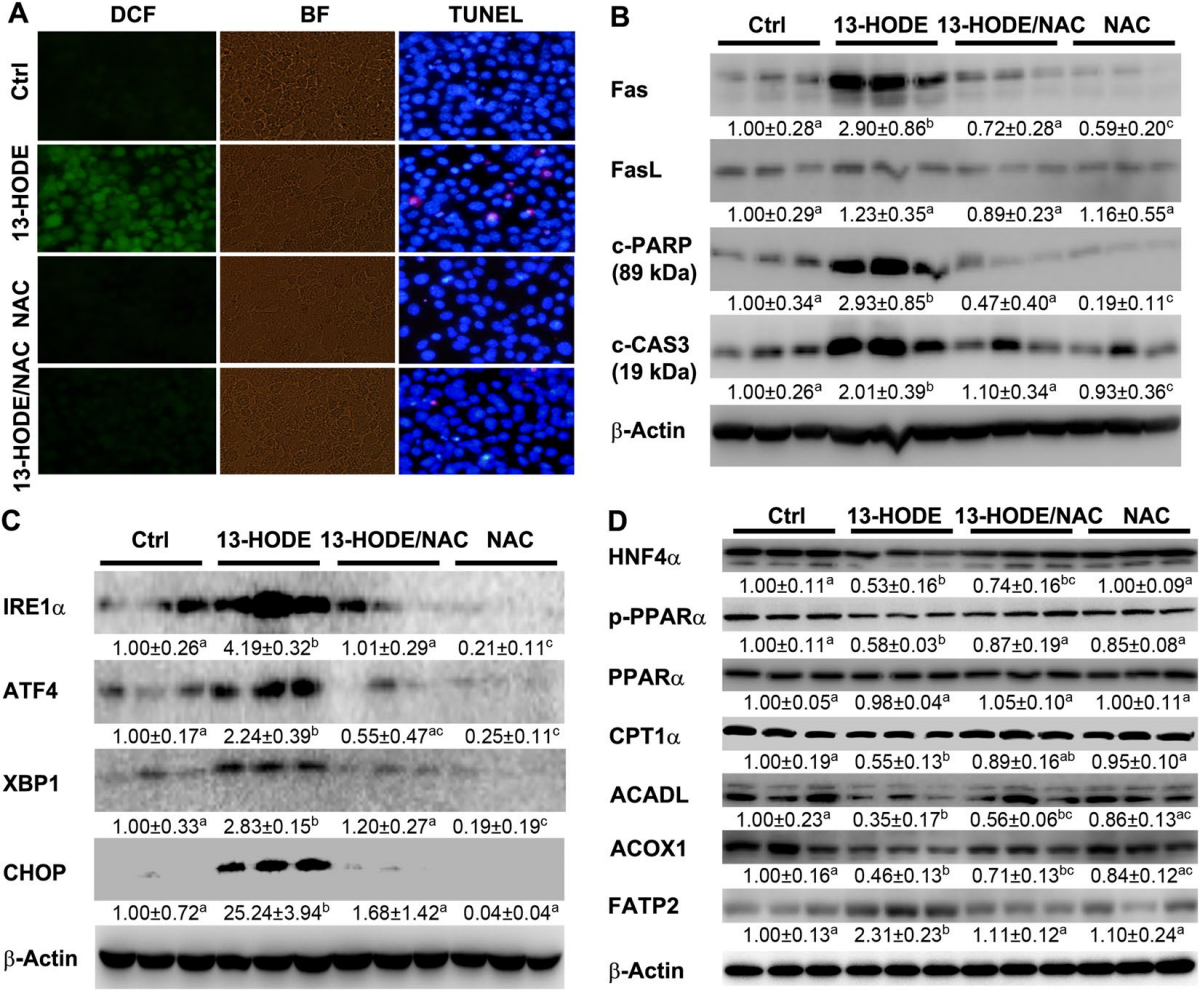
13-HODE induced ROS production, ER stress, perturbation of lipid metabolism, and apoptosis in Hepa-1c1c7 cells. Hepa-1c1c7 cells were treated with 13-HODE at 1 μM in the presence or absence of NAC at 10 mM for 3 days. (**A**) ROS production was analyzed by DCF staining (left panel) and apoptosis was analyzed by TUNEL staining (right panel). (**B**) Immunoblot of proteins involved in apoptosis. (**C**) Immunoblot of proteins involved in ER stress. (**D**) Immunoblot of proteins involved in lipid metabolism. Protein levels were quantitated by NIH image J. All values are denoted as means ± SD (n = 3). Significant differences were indicated by different letters (ANOVA, P < 0.05). DCF: 2′,7′-dichlorodihydrofluorescein diacetate, BF: bright field, TUNEL: terminal deoxynucleotidyl transferase dUTP nick end labeling.

To define the role of 13-HODE in liver damage, Hepa-1c1c7 cells were challenged by 13-HODE in the presence or absence of NAC, and ROS, ER stress, and apoptosis were measured. 13-HODE significantly increased ROS levels in association with induction of apoptosis. NAC significantly reduced 13-HODE-induced ROS. Accordingly, NAC also significantly inhibited 13-HODE-increased apoptosis of Hepa-1c1c7 cells (Fig. 2A, supplemental data). Protein levels of Fas, c-PARP, and c-CAS3 were significantly increased by 13-HODE compared to that of controls, which were normalized by NAC (Fig. 2B). Proteins levels of IRE1α, ATF4, XBP1, and CHOP were significantly increased by 13-HODE, but were normalized by NAC (Fig. 2C). Lipid metabolism regulators including HNF4α and p-PPARα, and fatty acid β–oxidation enzymes including CPT1α, ACADL and ACOX1 were significantly suppressed by 13-HODE, which was reversed by NAC (Fig. 2D).

### Linoleic acid is an inducer of ALOX15 expression in hepatocytes

**Figure 3:**
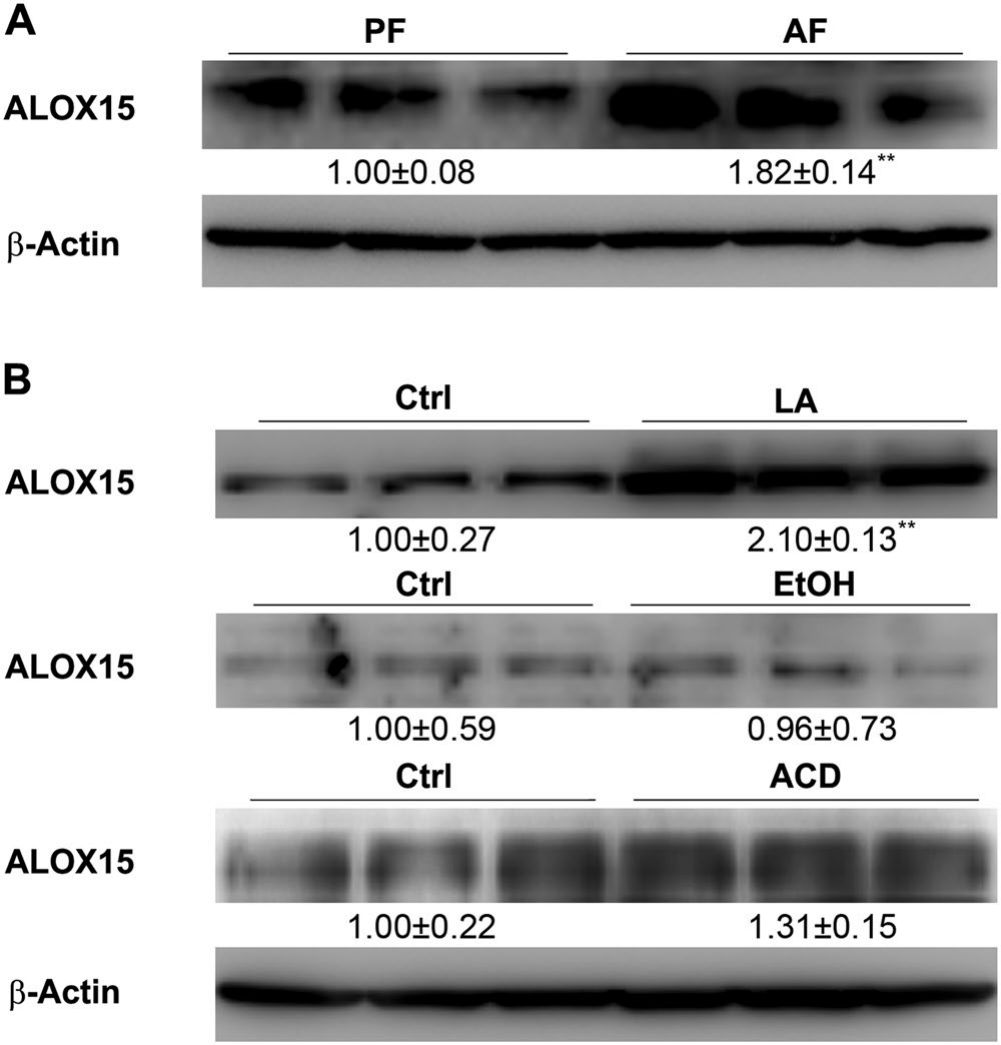
Linoleic acid induced ALOX15 expression. WT male mice were fed with control (PF) or alcohol (AF) liquid diet for 8 weeks. (**A**) Immunoblot of liver ALOX15. (**B**) Immunoblot of ALOX15 in Hepa-1c1c7 cells treated with linoleic acid, ethanol, or acetaldehyde. Protein levels were quantitated by NIH image J. All values are denoted as means ± SD (n = 3). **P < 0.01.

Protein levels of ALOX15, an inducible enzyme that plays a key role in the production of 13-HODE^21^, were measured by immunoblot. As shown in Fig. 3A, protein levels of hepatic ALOX15 were significantly increased by alcohol feeding. To investigate how alcohol exposure up-regulates ALOX15, Hepa-1c1c7 cells were treated with hepatic metabolites, which are increased by alcohol, including linoleic acid, ethanol, or acetaldehyde. Immunoblot results showed that ALOX15 were significantly increased by linoleic acid treatment, but not by ethanol or acetaldehyde (Fig. 3B).

### ALOX15 knockout ameliorates alcohol-induced ROS production, ER stress, apoptosis, and liver injury

**Figure 4:**
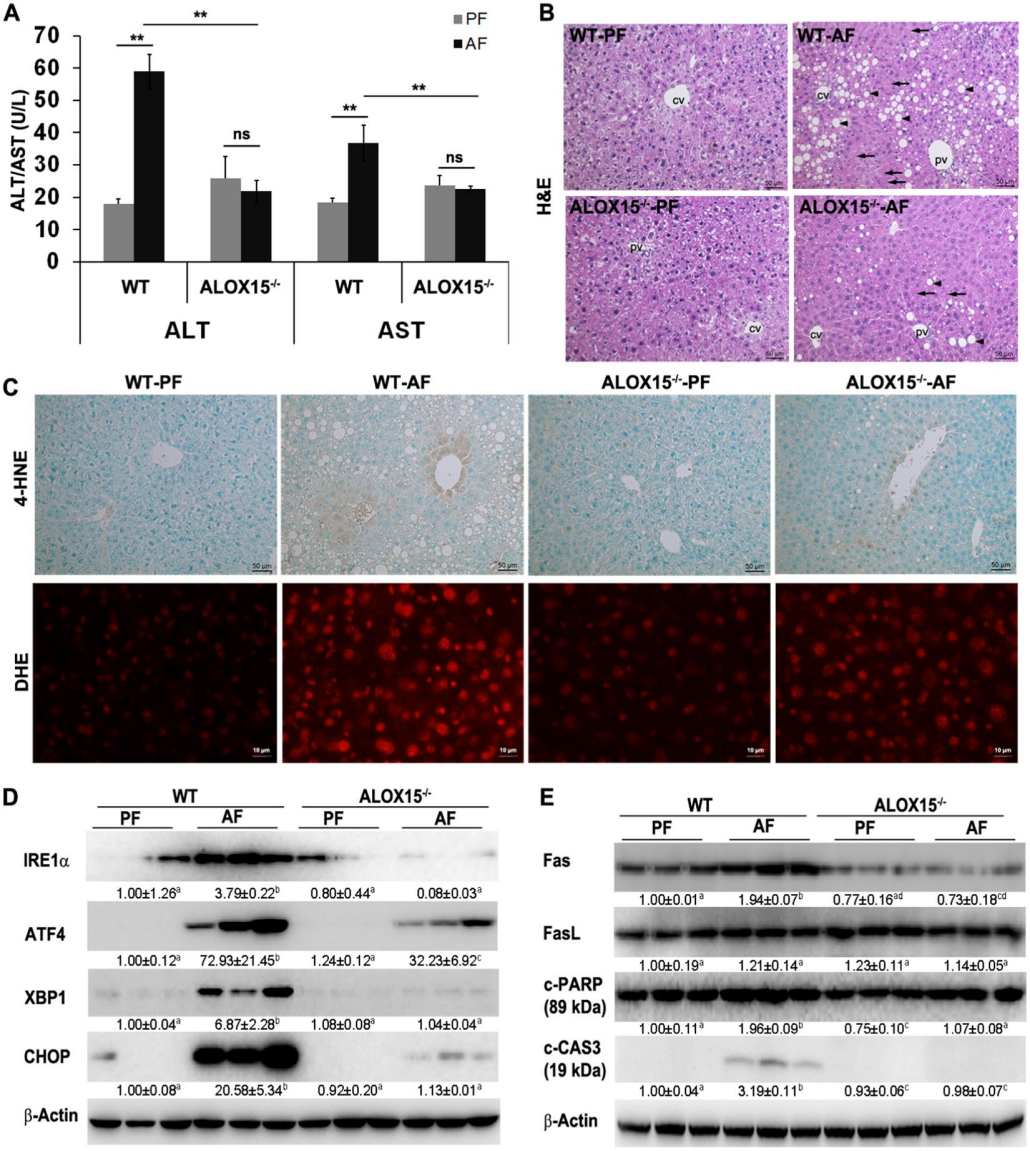
ALOX15 knockout attenuated alcohol-induced liver injury, ROS production, ER stress, and apoptosis. WT and ALOX15^−/−^ male mice were fed with control (PF) or alcohol (AF) liquid diet for 8 weeks. (**A**) Plasma ALT and AST. (**B**) Liver histopathological changes shown by H&E staining (arrows: necrosis, arrowheads: lipid droplets). (**C**) Representative images of 4-HNE staining (upper panel) and DHE staining (lower panel). (**D**) Immunoblot of proteins involved in ER stress. (**E**) Immunoblot of proteins involved in apoptosis. Protein levels were quantitated by NIH image J. All values are denoted as means ± SD (n = 6 in A, n = 3 in D and E). Significant differences were indicated by different letters (ANOVA, P < 0.05). *P < 0.05, **P < 0.01, ns, not significant (t-test). H&E: hematoxylin and eosin staining, 4-HNE: 4-hydroxynonenol, DHE: Dihydroethidium, CV: central vein, PV: portal vein.

ALOX15^−/−^ mice were used to define the role of ALOX15 in the development of ALD. As illustrated in Fig. 4, alcohol feeding significant increased plasma ALT and AST in WT mice, which was prevented in ALOX15^−/−^ mice (Fig. 4A). Knockout of ALOX15 also significantly diminished alcohol-induced liver histopathological changes, including necrosis and lipid accumulation (Fig. 4B). Alcohol feeding resulted in ROS production as indicated by both 4-HNE staining and DHE staining (Fig. 4C), which was significantly inhibited in ALOX15^−/−^ mice. ER stress markers including IRE1α, ATF4, XBP1 and CHOP were significantly up-regulated by alcohol in WT mice, but were significantly ameliorated in ALOX15^−/−^ mice (Fig. 4D). ALOX15 knockout also prevented alcohol-induced up-regulation of apoptotic signaling molecules including Fas, c-PARP, and c-CAS3 (Fig. 4E).

### ALOX15 knockout ameliorates alcohol-induced perturbations of lipid metabolism

**Figure 5:**
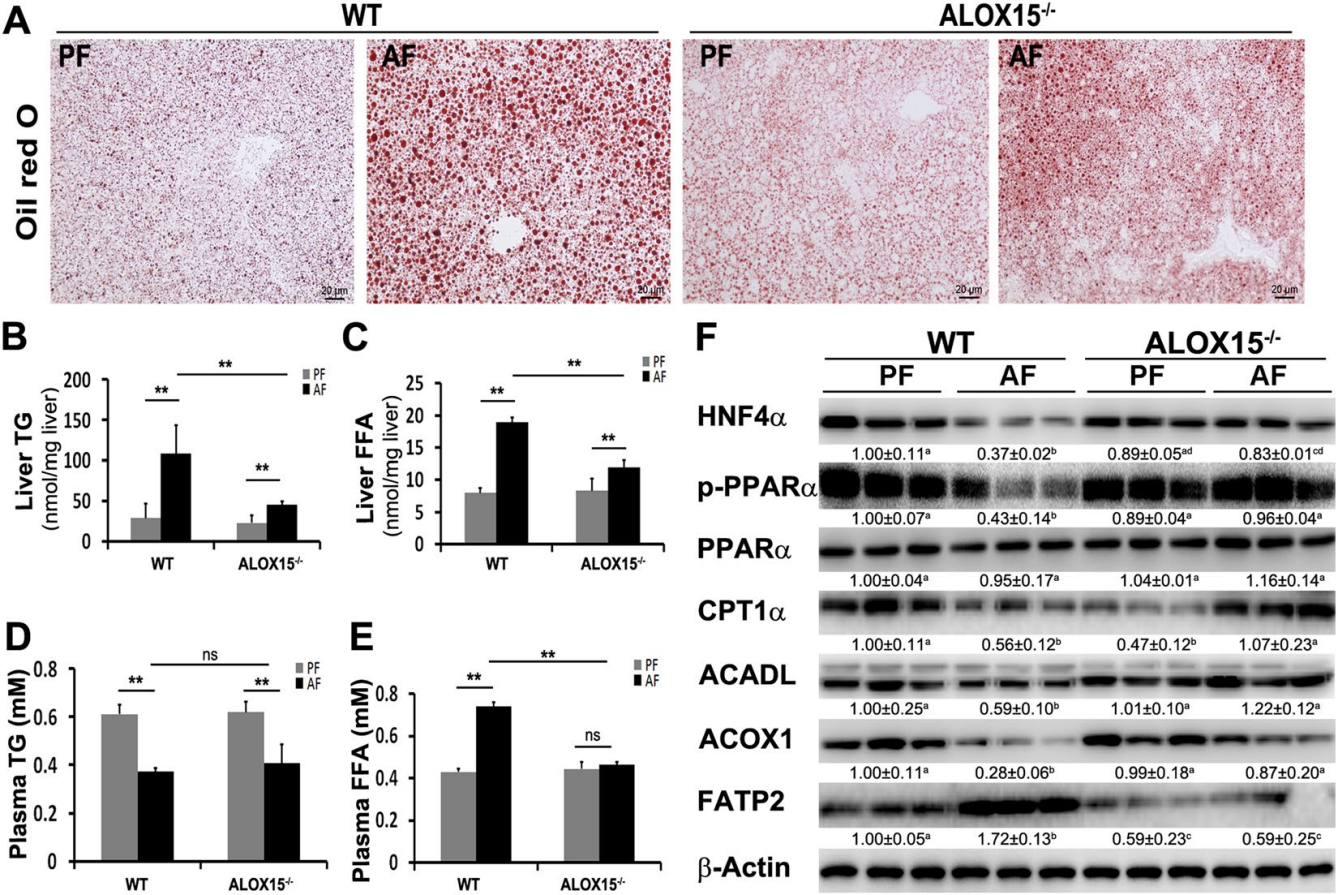
ALOX15 knockout attenuated alcohol-induced perturbation of lipid metabolism. WT and ALOX15^−/−^ male mice were fed with control (PF) or alcohol (AF) liquid diet for 8 weeks. (**A**) Representative image of Oil red O staining of liver sections. (**B**) Liver TG content. (**C**) Liver FFA concentration. (**D**) Plasma TG content. (**E**) Plasma FFA concentration. (**F**) Immunoblot of proteins involved in lipid metabolism. Protein levels were quantitated by NIH image J. All values are denoted as means ± SD (n = 6 in B-E, n = 3 in F). Significant differences were indicated by different letters (ANOVA, P < 0.05). *P < 0.05, **P < 0.01, ns, not significant (t-test). TG: triglyceride, FFA: free fatty acid.

Knockout of ALOX15 significantly ameliorated alcohol-induced lipid accumulation (Fig. 5A). Alcohol feeding significantly elevated levels of liver TG, liver FFA, and plasma FFA in WT mice, which were ameliorated in ALOX15^−/−^ mice (Fig. 5B,C and E). Knockout of ALOX15 showed no significant effect on plasma TG levels (Fig. 5D). Immunoblot results showed that in WT mice, alcohol feeding significantly reduced lipid metabolism regulators including HNF4α and p-PPARα and fatty acid oxidation related proteins including, CPT1α, ACOX1, and ACADL; all these alterations were attenuated in ALOX15^−/−^ mice. Knockout of ALOX15 also normalized alcohol-upregulated fatty acid transport protein, FATP2 (Fig. 5F).

### Administration of ALOX15 inhibitor, PD146176, alleviates alcohol-induced liver injury

**Figure 6:**
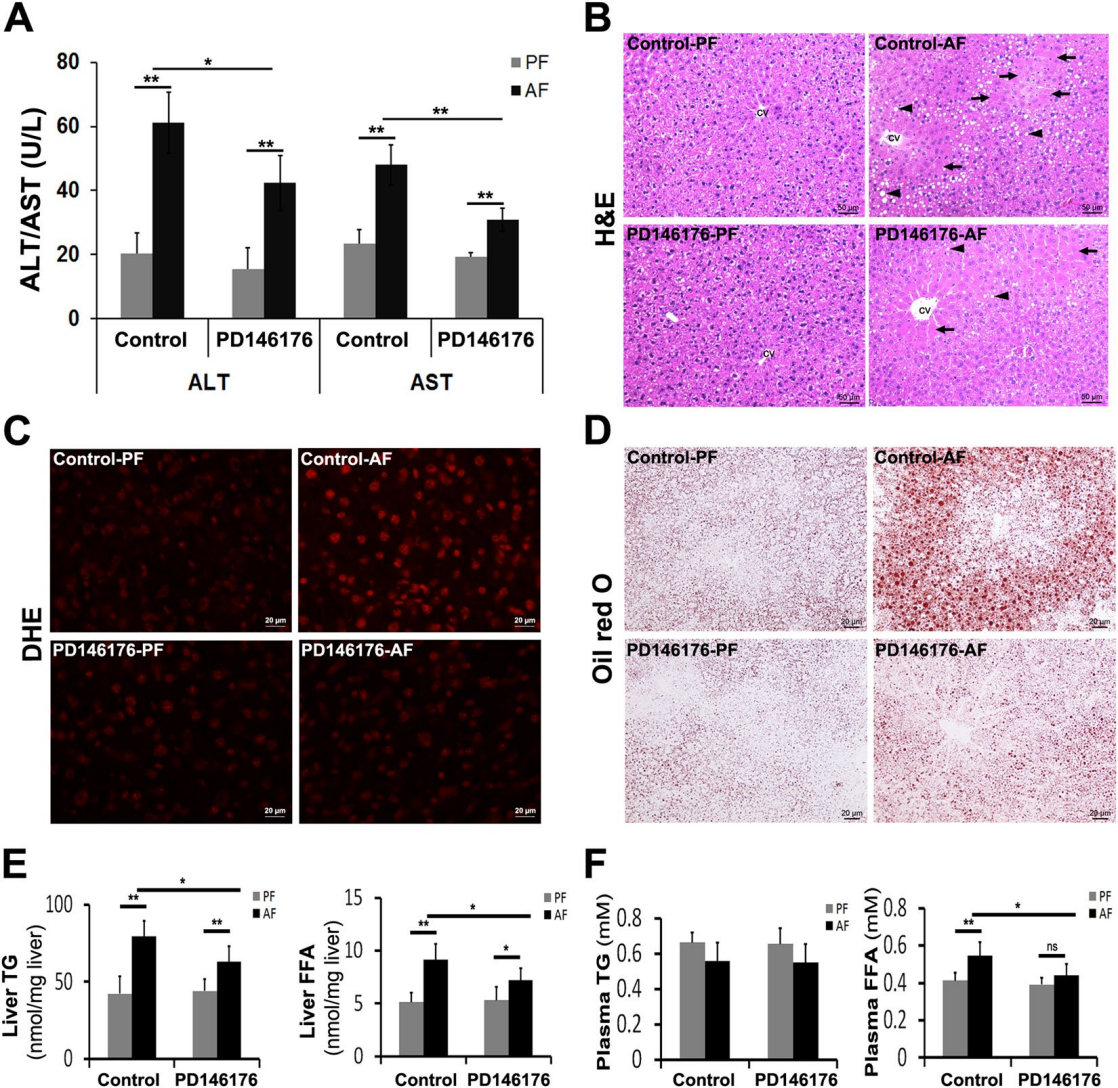
Administration of ALOX15 inhibitor, PD146176, significantly alleviated alcohol-induced hepatic injury in mice. WT male mice were fed with control (PF) or alcohol (AF) liquid diet for 2 weeks plus an oral gavage each week, and PD146176 was administrated once a day via i.p. injection for the second week. (**A**) Plasma ALT and AST. (**B**) Liver histopathological changes shown by H&E staining (arrows: necrosis, arrowheads: lipid droplets). (**C**) ROS production (DHE staining). (**D**) Oil red O staining of neutral lipids. (**E**) Liver TG and FFA concentration. (**F**) Plasma TG and FFA concentration. All values are denoted as means ± SD (n = 6 in A, and E-F). *P < 0.05, **P < 0.01, ns, not significant (t-test). DHE: Dihydroethidium, TG: triglyceride, FFA: free fatty acid, CV: central vein.

A clinically relevant acute-on-chronic alcohol exposure model was used to test the efficiency of ALOX15 inhibitor, PD146176, in treating ALD. PD146176 administration once a day for the last 7 days during 2 weeks of alcohol exposure significantly alleviated alcohol-elevated plasma AST and ALT (Fig. 6A), diminished alcohol-induced liver histopathological changes, including necrosis and lipid accumulation (Fig. 6B), inhibited alcohol-induced ROS production (Fig. 6C), and suppressed alcohol-induced hepatic lipid accumulation (Fig. 6D). Alcohol-elevated liver TG, liver FFAs, and plasma FFA levels were also significantly inhibited by administration of PD146176 (Fig. 6E,F).

## Discussion

The role of ALOX15/13-HODE in alcohol-induced liver injury was investigated in this study. Data showed that alcohol exposure significantly increased hepatic 13-HODE levels, induced ROS production, and promoted liver apoptosis. Mechanistically, we showed that administration of 13-HODE significantly induced ROS production, ER stress, perturbation of lipid metabolism, and apoptosis, which was diminished by neutralization of ROS with NAC, in Hepa-1c1c7 cells. Immunoblot analysis showed that chronic alcohol feeding significantly increased protein levels of 13-HODE-generating enzyme, ALOX15, in mouse liver. The induction of ALOX15 was also found in Hepa-1c1c7 cells treated by linoleic acid, which was increased by chronic alcohol feeding^5^. ALOX15 knockout protected mice from alcohol-induced liver injury. Inhibition of ALOX15 by its inhibitor, PD146176, significantly alleviated alcohol-induced liver injury.

It has been shown that apoptosis is a typical pathological feature of liver diseases including ALD. The causative factors of liver apoptosis include alcohol, viruses, toxic bile acids, fatty acids, drugs, and immune response^12, 22^. This study showed that chronic alcohol feeding promoted apoptosis (Fig. 1A). It is known that apoptosis can be initiated by both extrinsic and intrinsic pathways^23^. The significant induction of apoptotic proteins including Fas, c-CAS3, and c-PARP (Fig. 1B) by alcohol feeding indicated that alcohol exposure triggered extrinsic apoptotic pathway. Alcohol feeding has been shown to induce hepatocyte necrosis^24–26^. For example, Wang *et al*. has reported that Gao-binge alcohol treatment predominantly induces necrosis in mouse livers^24^. This study showed that ethanol-induced hepatocyte necrosis was significantly inhibited by knockout of ALOX15, suggesting that ALOX15/13-HODE plays a role in ethanol-induced hepatocyte necrosis and is a potential therapeutic target.

Chronic alcohol feeding has been shown to result in disruption of lipid homeostasis at WAT-liver axis, leading to increased lipid deposition in the liver^9, 27^. Our group reported that chronic alcohol feeding increased hepatic fatty acids levels, such as linoleic acid, arachidonic acid, and eicosadienoic acid^5^. But the effect of fatty acids metabolites on ALD remains elusive. This study showed that oxidized metabolites of linoleic acid, 13- and 9- HODE, were significantly increased by chronic alcohol feeding (Fig. 1C). HODEs have been shown to play an important role in inhibition of the intracellular calcium increase^28^, induction of vasodilatation^29^, suppression of proliferation and induction of apoptosis^30^. This study showed that 13-HODE induced ER stress, perturbed lipid metabolism, and enhanced protein levels of Fas, c-CAS3, and c-PARP, leading to apoptosis and suggested that 13-HODE plays an important role in the pathogenesis of ALD. The reason why there was no change in the concentrations of oxidized metabolites of arachidonic acid, HETEs, could be that HETEs were further metabolized into dihydroxyETEs (diHETEs) or other products^31, 32^.

Alcohol exposure has been shown to induce ROS production^33–35^. The present study showed that 13-HODE contributes to alcohol-induced ROS production. Moreover, neutralization of 13-HODE-induced ROS by NAC significantly inhibited 13-HODE-induced ER stress, alteration of lipid metabolism, and apoptosis, indicating that ROS played a critical role in 13-HODE-induced apoptosis (Fig. 2).

As mentioned earlier, ALOX15 plays a key role in the production of 13-HODE from linoleic acid^21^. The increase of hepatic 13-HODE level led us to investigate whether alcohol exposure affected ALOX15 expression. Data showed that alcohol significantly up-regulated ALOX15 in mouse liver (Fig. 3A), accounting for the over-production of 13-HODE. To investigate how alcohol exposure induces ALOX15, ethanol, linoleic acid, or acetaldehyde was used to treat Hepa-1c1c7 cells. Immunoblot results showed that linoleic acid, which has been shown elevated by alcohol-feeding^5^, significantly induced ALOX15 expression, while ethanol and acetaldehyde showed no significant effect on ALOX15 expression (Fig. 3), suggesting that alcohol-induced increase of linoleic acid accounts for the up-regulation of ALOX15 and subsequent increase of 13-HODE. It is known that linoleic acid is converted by various lipoxygenases, cyclooxygenases, certain cytochrome P450 enzymes, and non-enxymatic autooxidation mechanisms^36^. The fact that ethanol or acetaldehyde did not affect ALOX suggested that ethanol or acetaldehyde did not alter the balance of lipoxygenases, cyclooxygenases, certain cytochrome P450 enzymes, and non-enxymatic autooxidation mechanisms in Hepa-1c1c7 cells. We are interested to investigate the mechanism by which alcohol exposure affects linoleic acid metabolism and ALOX15 expression using different types of cells in future study.

To further elucidate the role of ALOX15/13-HODE signaling in the progression of ALD, ALOX15^−/−^ mice were introduced. ALOX15 knockout significantly attenuated alcohol-induced elevation of ALT and AST, alleviated liver injury, inhibited ROS production, suppressed ER stress, and abolished alcohol-induced apoptosis (Fig. 4). ALOX15 knockout significantly lowered the number and size of lipid droplets, hepatic TG level, hepatic FFA level, and plasma FFA level, compared to that of WT mice. In addition, knockout of ALOX15 reversed alcohol-induced perturbation of lipid metabolism (Fig. 5). ALOX15 has been shown to be linked with nonalcoholic fatty liver disease and high fat diet-induced steatohepatitis^18, 19^, the present study suggests that ALOX15/13-HODE signaling promoted alcohol-induced hepatic steatosis, ROS production, ER stress, alteration of lipid metabolism, and apoptosis, and knockout of ALOX15 protected mice against alcohol-induced hepatic injury.

PD146176 is a specific inhibitor of ALOX15 which has been shown to limit monocyte macrophage enrichment of atherosclerotic lesions and attenuate development of fibrofoamy and fibrous plaque lesions^37, 38^. This study showed administration of PD146176 protected mice against alcohol-induced liver damage by down-regulation of alcohol-enhanced AST/ALT activity, suppression of ROS production, alleviation of alcohol-induced liver steatosis, and decrease of alcohol-elevated levels of hepatic TG, hepatic FFA, and plasma FFA (Fig. 6). To our knowledge, this is the first report of the therapeutic effects of PD146176 on alcohol-induced liver injury.

## Conclusions

In summary, the present study demonstrated that chronic alcohol feeding increased hepatic 13-HODE level via up-regulation of ALOX15 by linoleic acid. Increased 13-HODE contributed to alcohol-induced generation of ROS, ER stress, alteration of lipid metabolism, and activation of apoptotic pathway in the liver. Administration of ALOX15 inhibitor reversed alcohol-induced liver injury. This study suggests that ALOX15 is a potential therapeutic target for treatment of ALD.

## Materials and Methods

### Animals and alcohol feeding

Male C57BL/6 J wild type (WT) and ALOX15^−/−^ mice were obtained from the Jackson Laboratory (Bar Harbor, ME). The animal protocol was approved by the Institutional Animal Care and Use Committee of the North Carolina Research Campus (16-017). Experimental procedures were carried out in accordance with all regulations regarding the care and use of experimental animals (National Institutes of Health).

### Animal study 1

To determine the effects of ethanol exposure on liver injury and polyunsaturated fatty acids (PUFA) peroxidation derivatives, ten-week-old male WT mice were fed with the Lieber-DeCarli alcohol (alcohol-fed, AF) or control (pair-fed, PF) diet (Dyets, Bethlehem, PA) for 8 weeks (n = 8) and tissues were collected. The ethanol content (%, w/v) in the diet was started at 2.1 and gradually increased to 4.8%. The amount of food given to PF mice was that the AF mice consumed in the previous day. Mice were anesthetized with inhalational isoflurane and tissues were collected.

### Animal study 2

Ten-week-old male WT and ALOX15^−/−^ mice were pair-fed with the Lieber-DeCarli alcohol or control diet for 8 weeks as described in Animal study 1 (n = 8).

### Animal study 3

A clinically relevant acute-on-chronic alcohol exposure model was used to test the therapeutic potential of ALOX15 inhibitor, PD146176. Ten-week-old male C57BL/6 J mice were pair-fed with the Lieber-DeCarli alcohol (4% ethanol, w/v) or control diet for 2 weeks with one ethanol oral gavage at 4 g/kg body weight each week (n = 8). PD146176 (Tocris, Avonmouth, Bristol, UK), a specific ALOX15 inhibitor, was dissolved in DMSO, diluted with PBS, and given to mice at a dose of 10 mg/kg body weight (in a final volume of 300 µl) once a day for the second week via intraperitoneal injection. Control mice were given same amount of DMSO.

### Cell culture and treatments

Hepa-1c1c7 cells (ATCC, Rockville, MD, USA) were cultured in Alpha minimum essential medium without nucleosides (Life technologies, Grand Island, NY, USA) containing 10% fetal bovine serum (Atlanta Biologicals, Lawrenceville, GA), 100 U/mL penicillin and 100 μg/mL streptomycin (Invitrogen, Carlsbad, CA), at 37 °C in a humidified atmosphere of 5% CO_2_. 13-HODE (Cayman, Ann Arbor, MI) was used to treat Hepa-1c1c7 cells at 1 μM in the presence or absence of the antioxidant, N-acetyl-*L*-cysteine (NAC), at 10 mM for 3 days. Linoleic acid, ethanol, or acetaldehyde was also used to treat Hepa-1c1c7 cells at 200 μM, 100 mM, or 100 μM, respectively, for 3 days. Cells were washed twice with cold PBS and collected.

### LC-MS analysis of HODEs and HETEs

Samples (0.85 ml) were spiked with 5 ng each (in 150 µl methanol) of 15(S)-HETE-d8, Leukotriene B4-d4, and Prostaglandin E1-d4 as internal standards for recovery and quantitation and mixed thoroughly. The samples were then extracted for PUFA metabolites using C18 extraction columns as described earlier^39–41^. HPLC was performed on a Prominence XR system (Shimadzu) using Luna C18 (3 µ, 2.1 × 150 mm) column. The mobile phase consisted of a gradient between A: methanol-water-acetonitrile (10:85:5 v/v) and B: methanol-water-acetonitrile (90:5:5 v/v), both containing 0.1% ammonium acetate. The gradient program with respect to the composition of B was as follows: 0–1 min, 50%; 1–8 min, 50–80%; 8–15 min, 80–95%; and 15–17 min, 95%. The flow rate was 0.2 ml/min. The HPLC eluate was directly introduced to ESI source of QTRAP5500 mass analyzer (ABSCIEX) in the negative ion mode with following conditions: Curtain gas: 35 psi, GS1: 35 psi, GS2: 65 psi, Temperature: 600 °C, Ion Spray Voltage: −1500 V, Collision gas: low, Declustering Potential: −60 V, and Entrance Potential: −7 V. The eluate was monitored by Multiple Reaction Monitoring method to detect unique molecular ion – daughter ion combinations for each of the 125 transitions (to monitor a total of 144 lipid mediators) with 5 msec dwell time for each transition. Optimized Collisional Energies (18–35 eV) and Collision Cell Exit Potentials (7–10 V) were used for each MRM transition. The data was collected using Analyst 1.5.2 software and the MRM transition chromatograms were quantitated by MultiQuant software (both from ABSCIEX). The internal standard (15-HETE-d8) signal in each chromatogram was used for normalization for recovery as well as relative quantitation of each analyte.

### Oil red O staining

Livers were frozen in Tissue-Tek Optimum Cutting Temperature (OCT) Compound (VWR, Batavia, IL). Cryostat liver sections were cut at 5 µm, fixed with 10% formalin and stained with Oil red O solution.

### Histopathological analysis of liver

Livers were fixed in 10% formalin, and processed for paraffin embedding. Paraffin sections were cut at 5 µm and stained with hematoxylin and eosin (H&E) to assess the histological changes.

### Alanine aminotransferase (ALT) and aspartate aminotransferase (AST)

The ALT and AST activities in the serum were measured with an Infinity kit according to manufacturer’s instruction (Thermo Scientific, Waltham, MA).

### Quantitative assay of triglycerides and free fatty acids (FFAs)

The levels of triglycerides in liver and plasma were measured with assay kits according to manufacturer’s instruction (Thermo Scientific, Waltham, MA). Concentrations of FFAs in liver and plasma were determined with a FFA quantification kit (BioVision, Milpitas, CA).

### Immunoblot analysis

Whole protein lysates of livers and Hepa-1c1c7 cells were extracted using 10% Nonidet P-40 lysis buffer supplemented with protease inhibitor and phosphatase inhibitor (Sigma-Aldrich, St. Louis, MO). Fifty µg of proteins were loaded onto 8–12% SDS-PAGE, trans-blotted onto PVDF membrane, blocked with 5% nonfat milk in TBST for 1 hour, and incubated with anti-ALOX15, X-box binding protein 1 (XBP1), C/EBP homologous protein (CHOP), hepatocyte nuclear factor 4α (HNF4α) (Novus Biologicals, Littleton, CO, USA), Fas receptor (Fas), β-actin (Santa Cruz Biotechnology, Inc., Santa Cruz, CA), phospo-peroxisome proliferator-activated receptor α (p-PPARα), PPARα (Thermo Scientific, Waltham, MA), long chain acyl CoA dehydrogenase (ACADL), acyl CoA oxidase 1 (ACOX1), fatty acid transport protein 2 (FATP2), carnitine palmitoyltransferase 1 A (CPT1α) (Proteintech, Rosemont, IL), Fas ligand (FasL), cleaved poly ADP ribose polymerase (c-PARP), cleaved Caspase 3 (c-CAS3), inositol-requiring enzyme-1α (IRE1α), activating transcription factor 4 (ATF4) antibody (Cell Signaling Technology, Danvers, MA), respectively. Membranes were washed and incubated with horseradish peroxidase–conjugated secondary antibodies (Thermo Scientific, Rockford, IL, USA). Bound complexes were detected via enhanced chemiluminescence (GE Healthcare, Piscataway, NJ, USA). Bands were quantified, and the ratio to β-actin was calculated and given as fold changes, setting the values of pair-fed or control at 1.

### Immunohistochemistry

Liver sections were rehydrated and incubated with anti-4-hydroxynonenol (4-HNE) antibody (Northwest Life Science Specialties, Vancouver, WA) overnight at 4 °C, followed by incubation with a horseradish peroxidase-conjugated secondary antibody (Thermo Scientific) for 30 min at room temperature. Visualization was conducted using diaminobenzidine as HRP substrate.

### ROS measurement

The 2′,7′-dichlorodihydrofluorescein diacetate (DCF) (Life technologies, Grand Island, NY, USA) was used to measure ROS in Hepa-1c1c7 cells. Cells treated with 13-HODE at 1 μM for 3 days in the presence or absence of NAC at 10 mM were incubated with 20 μM DCF at 37 °C for 30 min in dark. Images were captured immediately with a fluorescent microscope.

Dihydroethidium (DHE) fluorescent microscopy was performed to detect superoxide on frozen liver sections. Cryostat liver sections were cut in 5 μm and incubated with 5 μM DHE (Life technologies, Carlsbad, CA) for 10 min at room temperature.

### Detection of apoptosis by TUNEL staining

The terminal deoxynucleotidyl transferase dUTP nick end labeling (TUNEL) assay kit (EMD Millipore, Billerica, MA) was used to detect DNA fragmentation according to manufacturer’s instruction. 13-HODE was used to treat Hepa-1c1c7 cells at 1 μM in the presence or absence of NAC, at 10 mM for 3 days. Cell slides were fixed with 1% paraformaldehyde for 10 min. Liver sections or cell slides were pretreated with proteinase K and H_2_O_2_ and incubated with the reaction mixture containing terminal deoxynucleotidyl transferase and digoxigenin-conjugated dUTP for 1 h at 37 °C. The labeled DNA was visualized with Alexa Fluor 594-conjugated anti-digoxigenin antibody (Jackson Immunoresearch, West Grove, PA) for cell slides or HRP-conjugated anti-digoxigenin antibody with diaminobenzidine as the chromagen for liver sections.

### Statistical analysis

Data are expressed as mean ± standard deviation (SD). Results were analyzed using the one-sample *t*-*test* or one-way analysis of variance (ANOVA) followed by Turkey’s HSD. In all tests, P < 0.05 was considered statistically significant.

## Electronic supplementary material

Supplementary Information
