# Supplementary material for: Hepatic overproduction of 13-HODE due to ALOX15 upregulation contributes to alcohol-induced liver injury in mice
Source: Sci Rep. 2017 Aug 21;7:8976. doi: 10.1038/s41598-017-02759-0 (PMC5567196; doi:10.1038/s41598-017-02759-0)

Hepatic overproduction of 13-HODE due to ALOX15 upregulation contributes to alcohol-induced liver injury in mice

Wenliang Zhang[1], Wei Zhong[1], Qian Sun[1], Xinguo Sun[1], Zhanxiang Zhou[1,2,*]

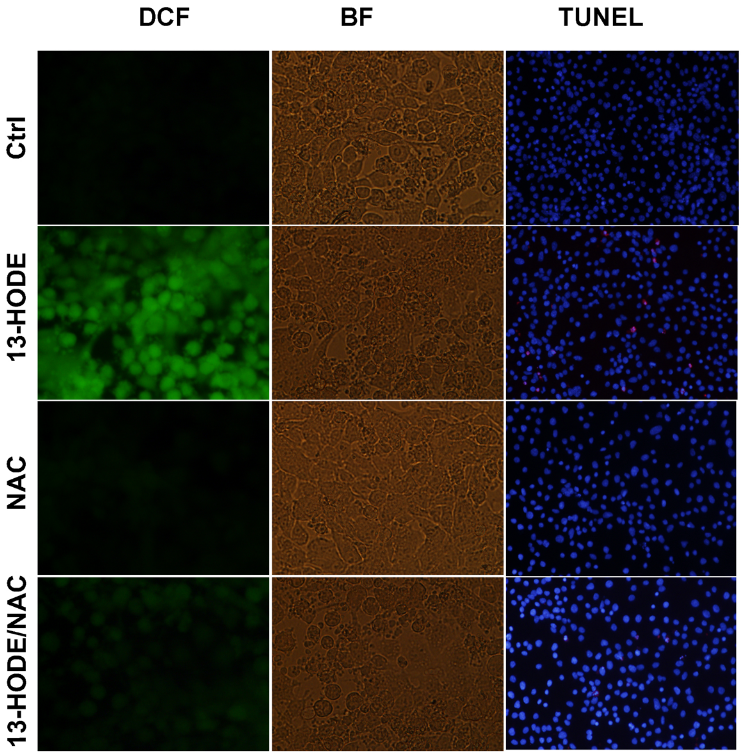
DCF
BF
TUNEL
Ctrl
13-HODE
NAC
13-HODE/NAC

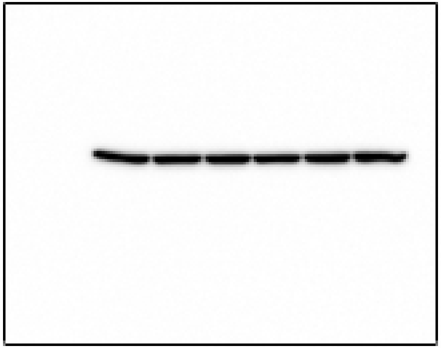

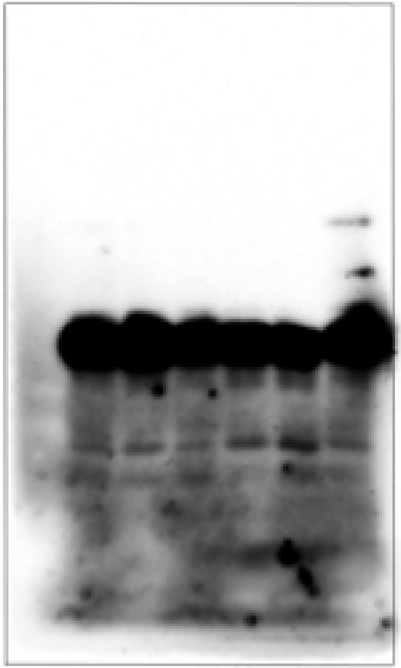

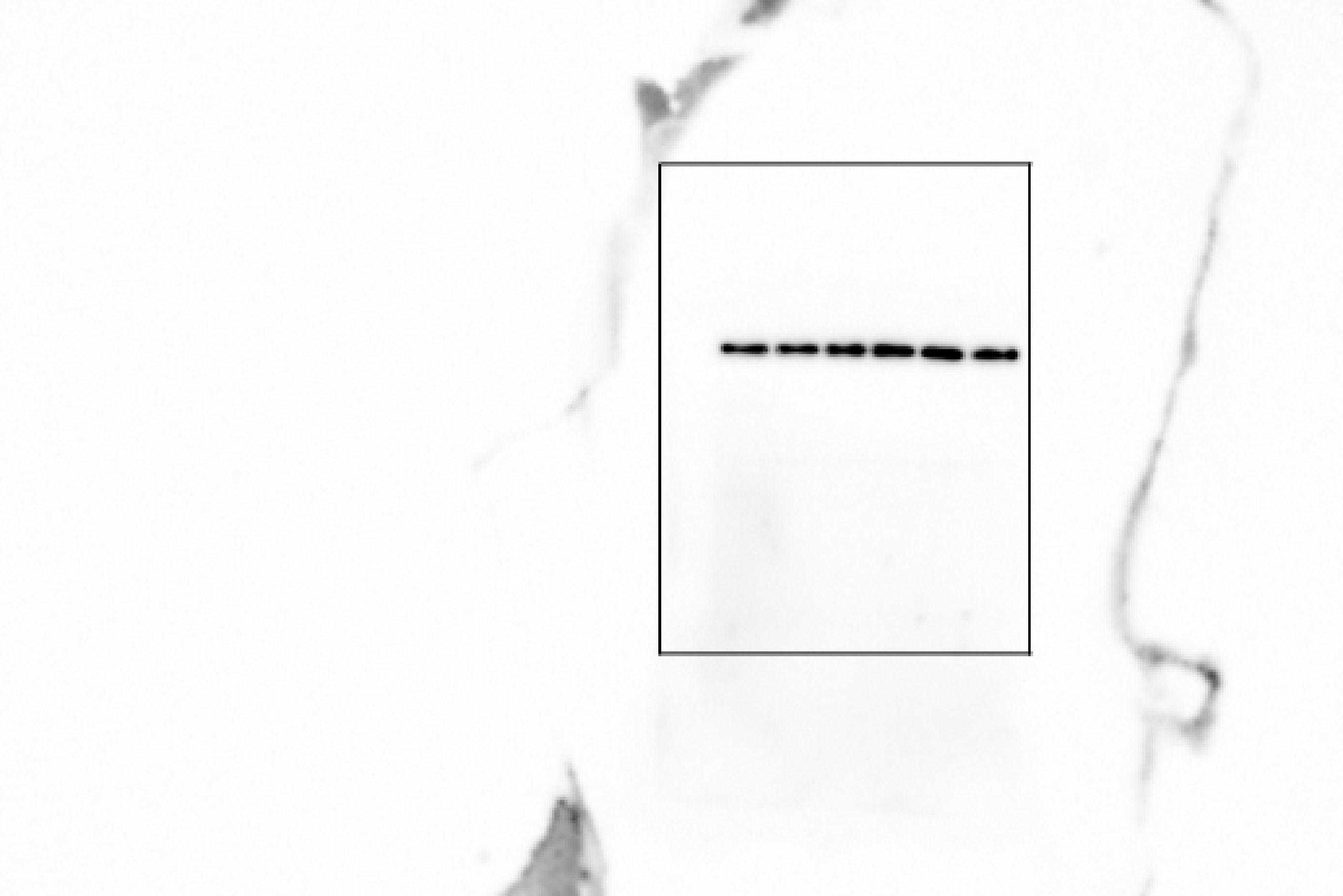

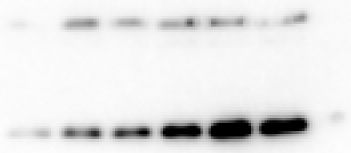

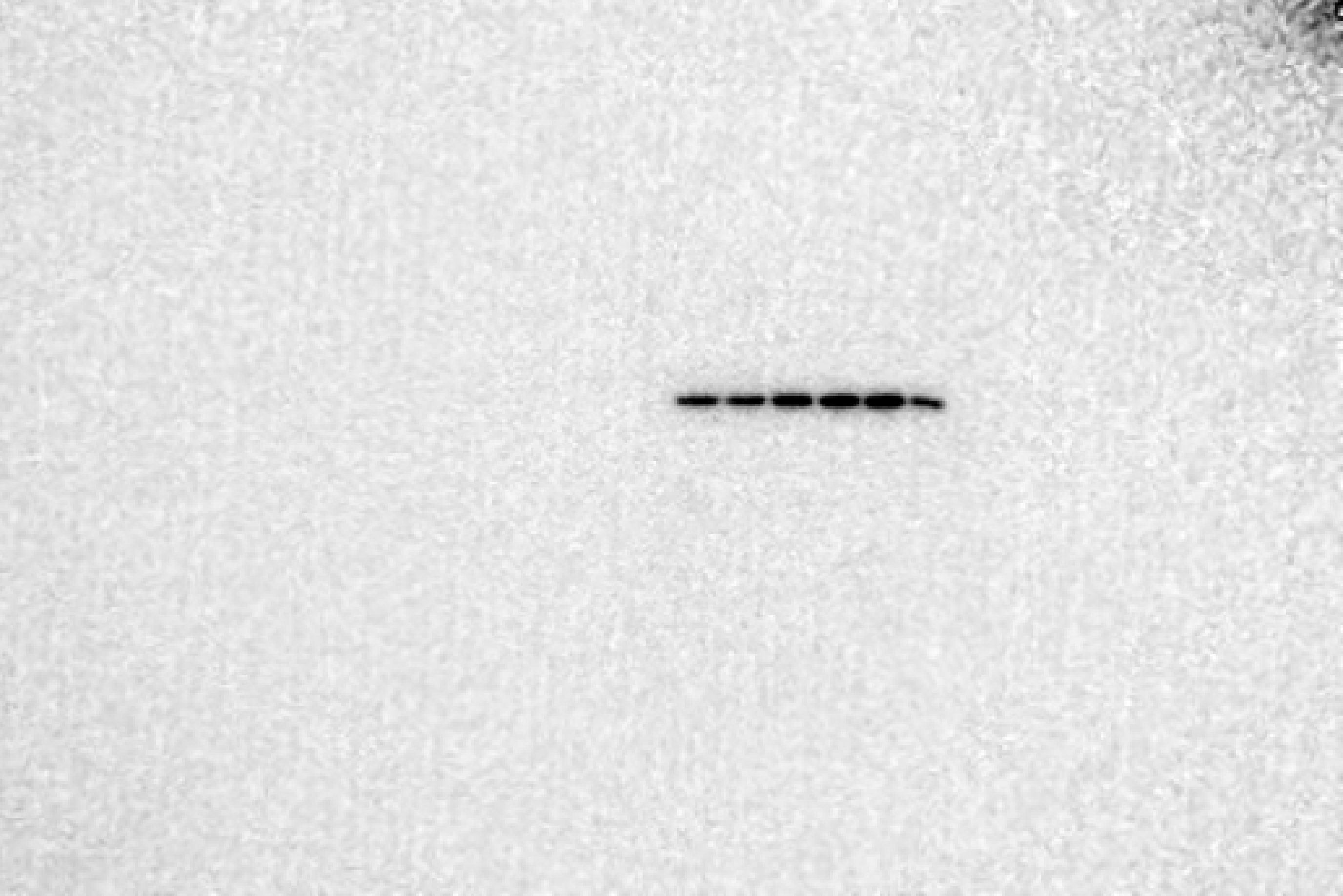

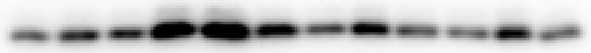

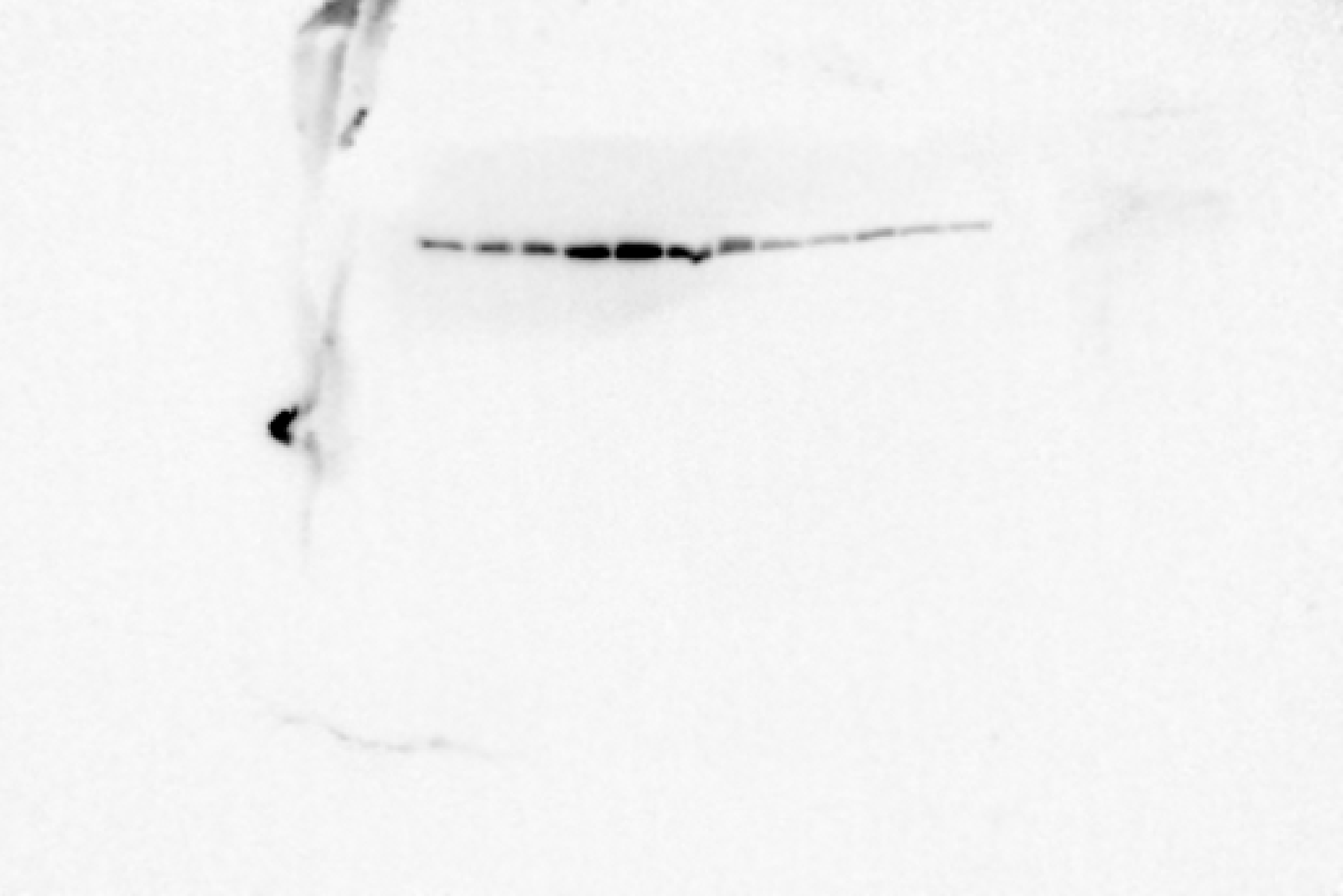

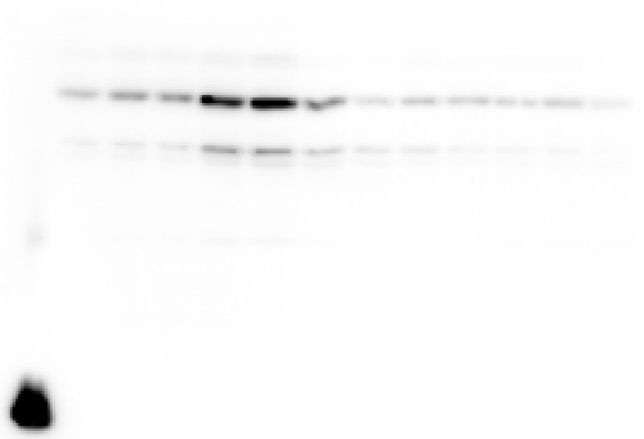

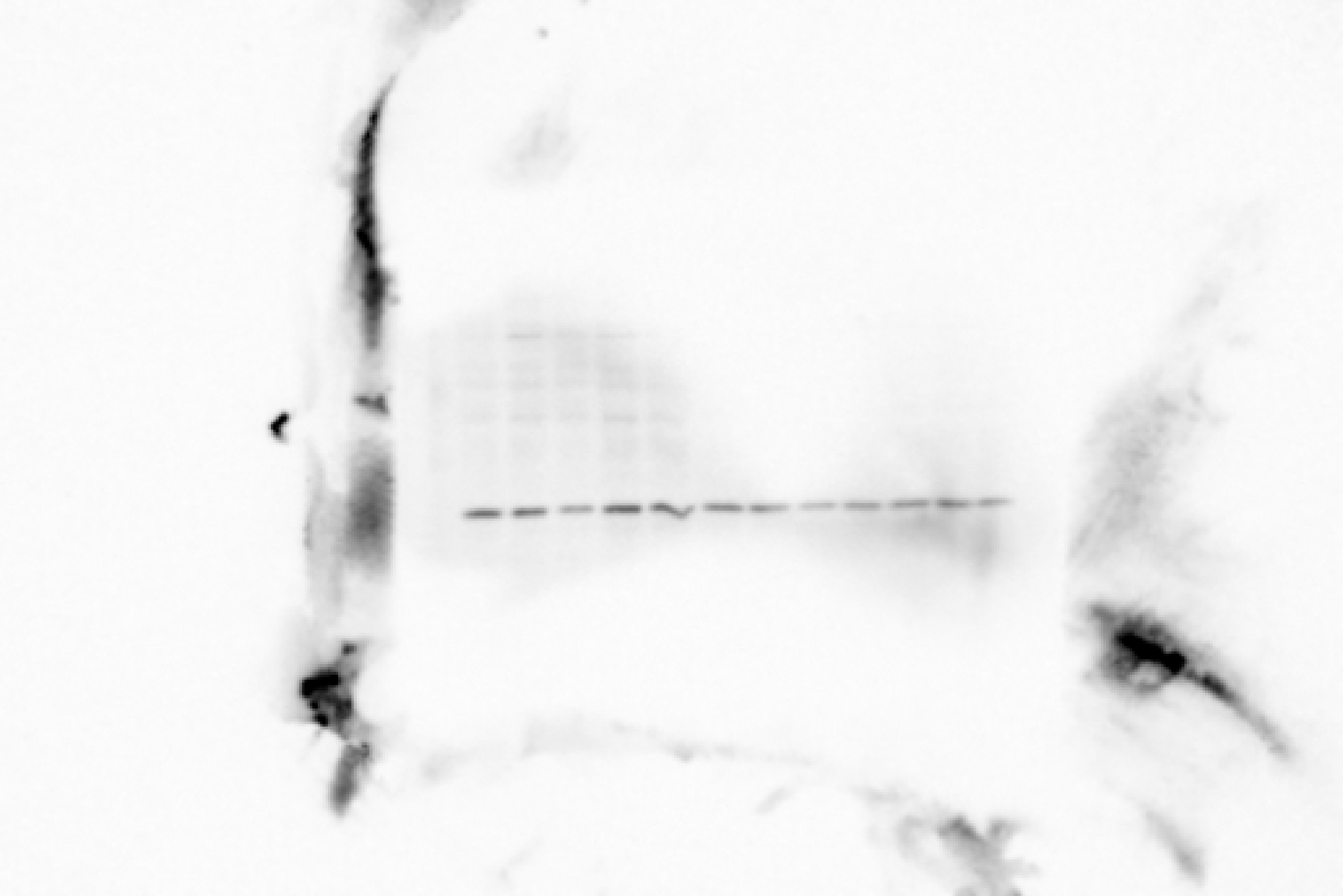

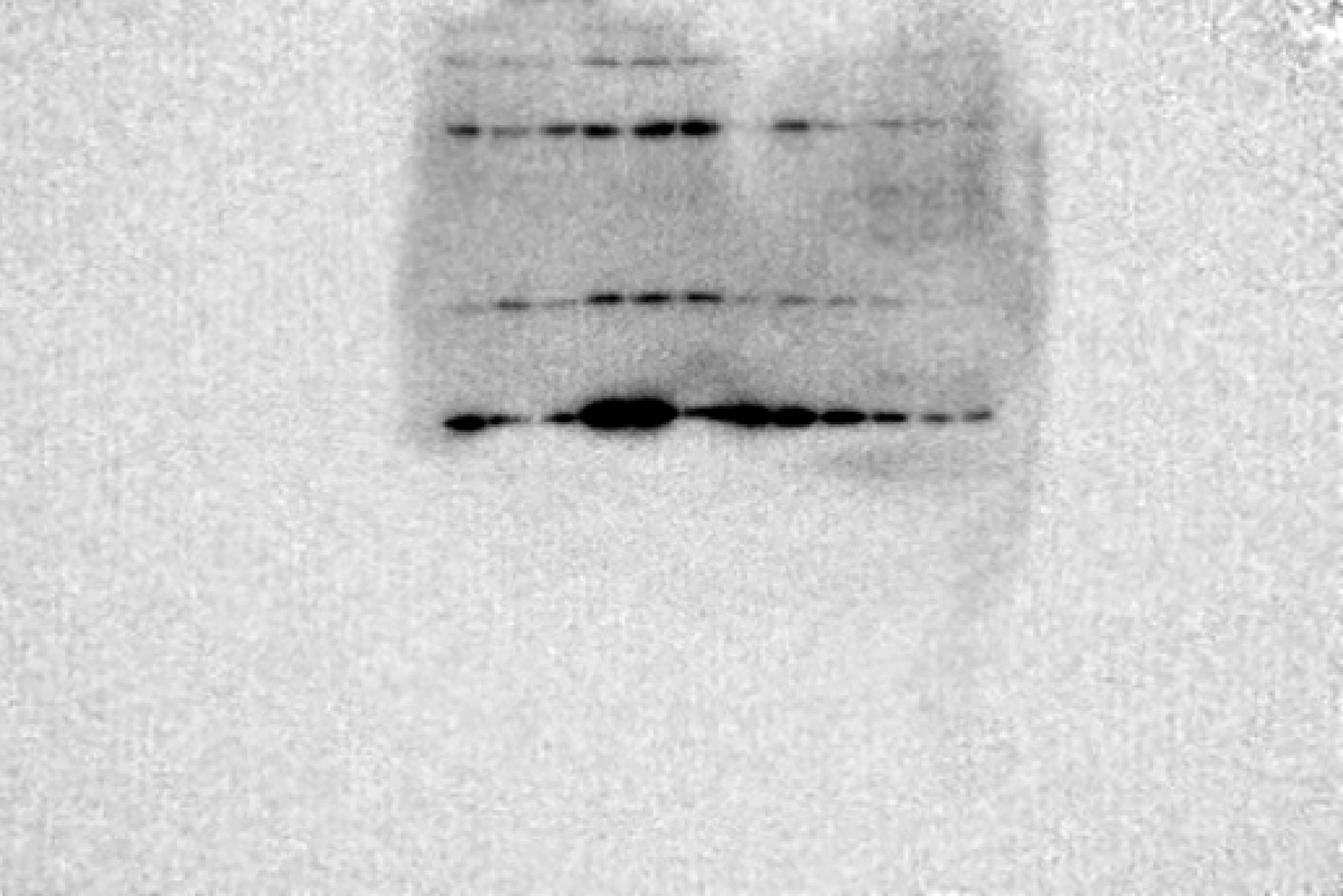

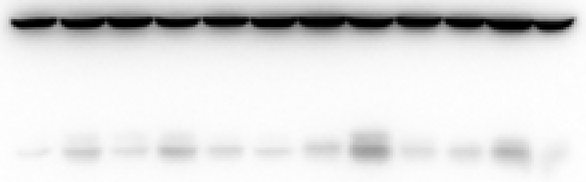

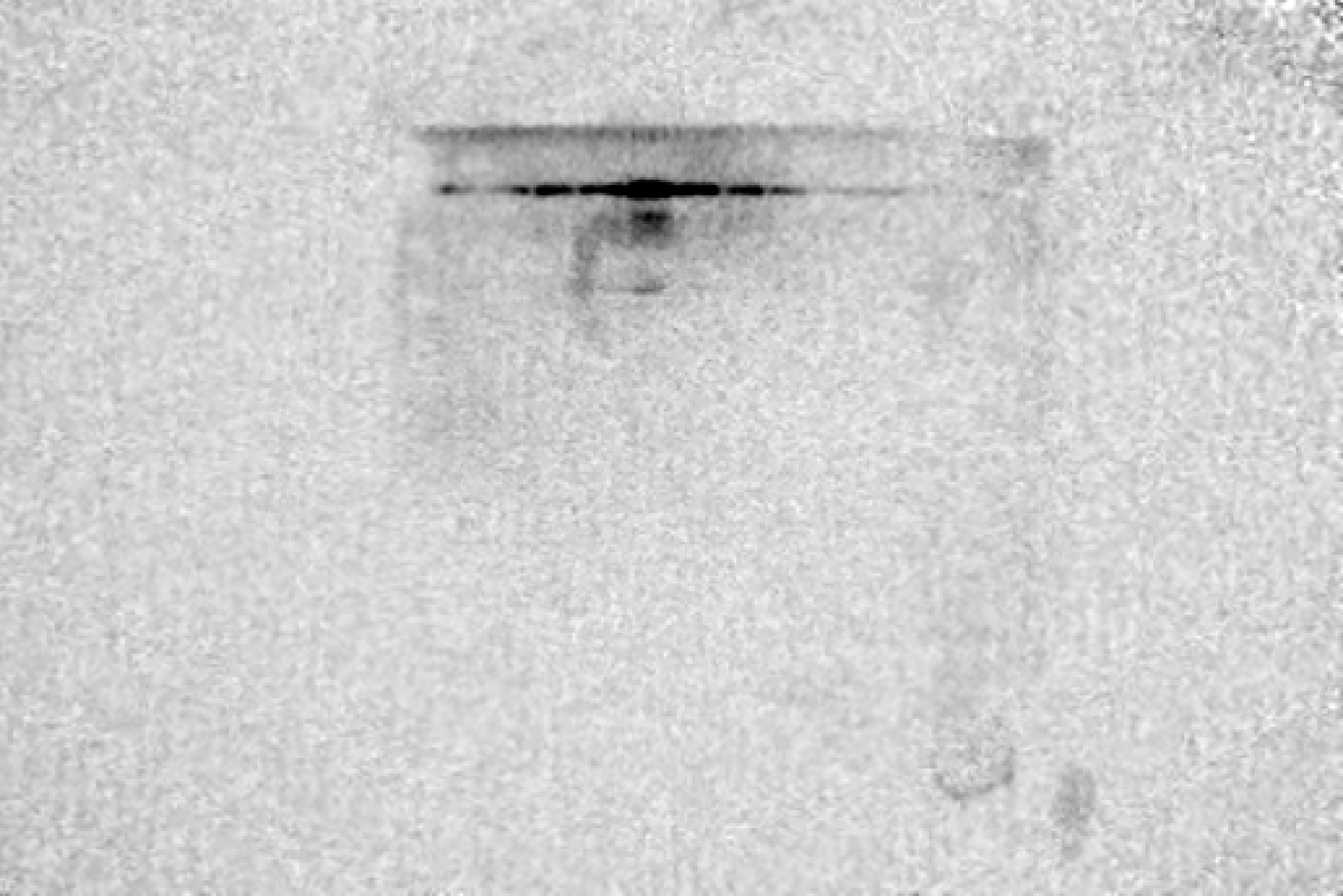

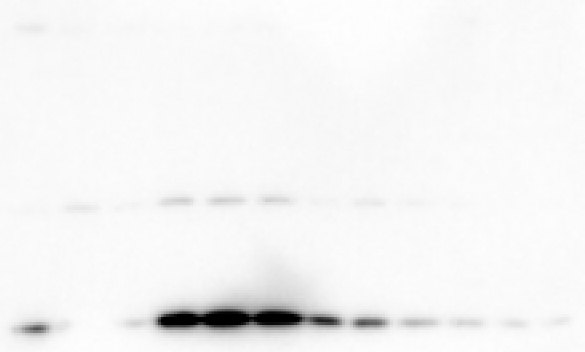

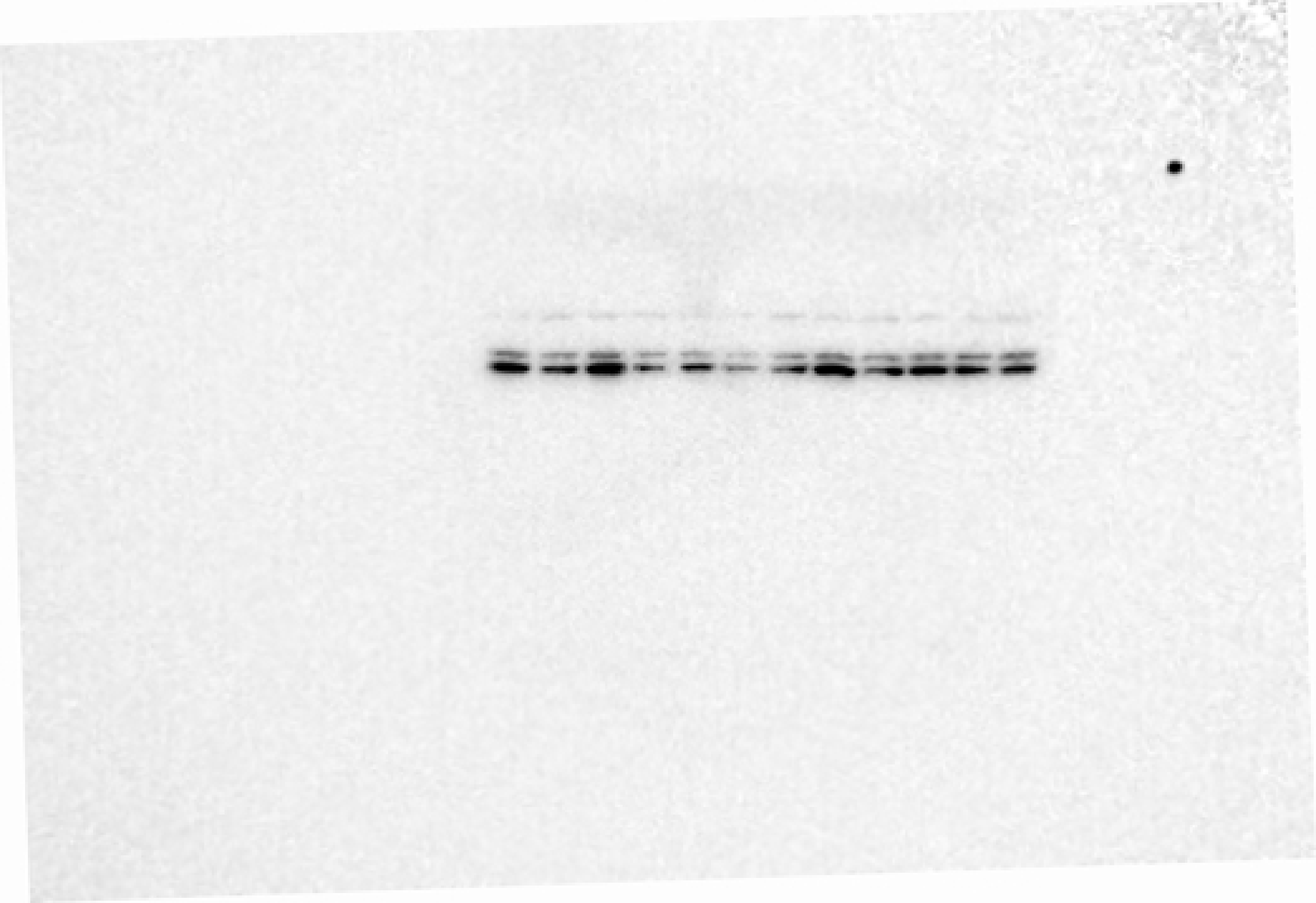

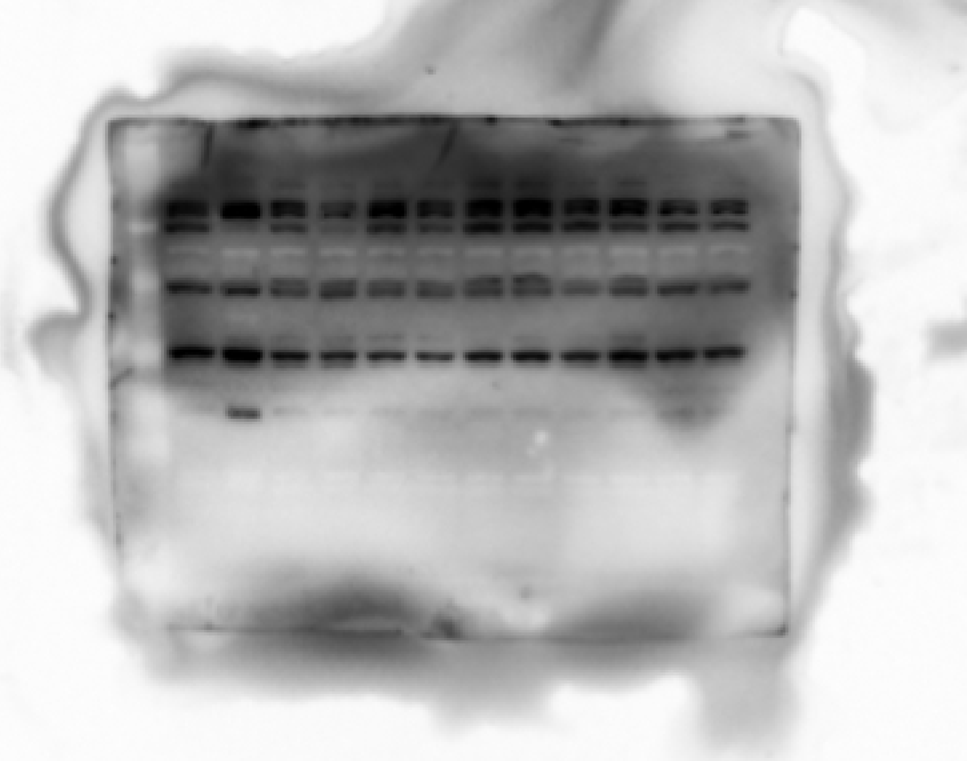

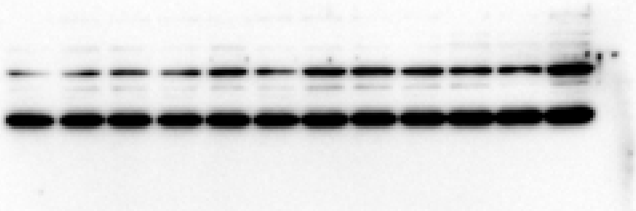

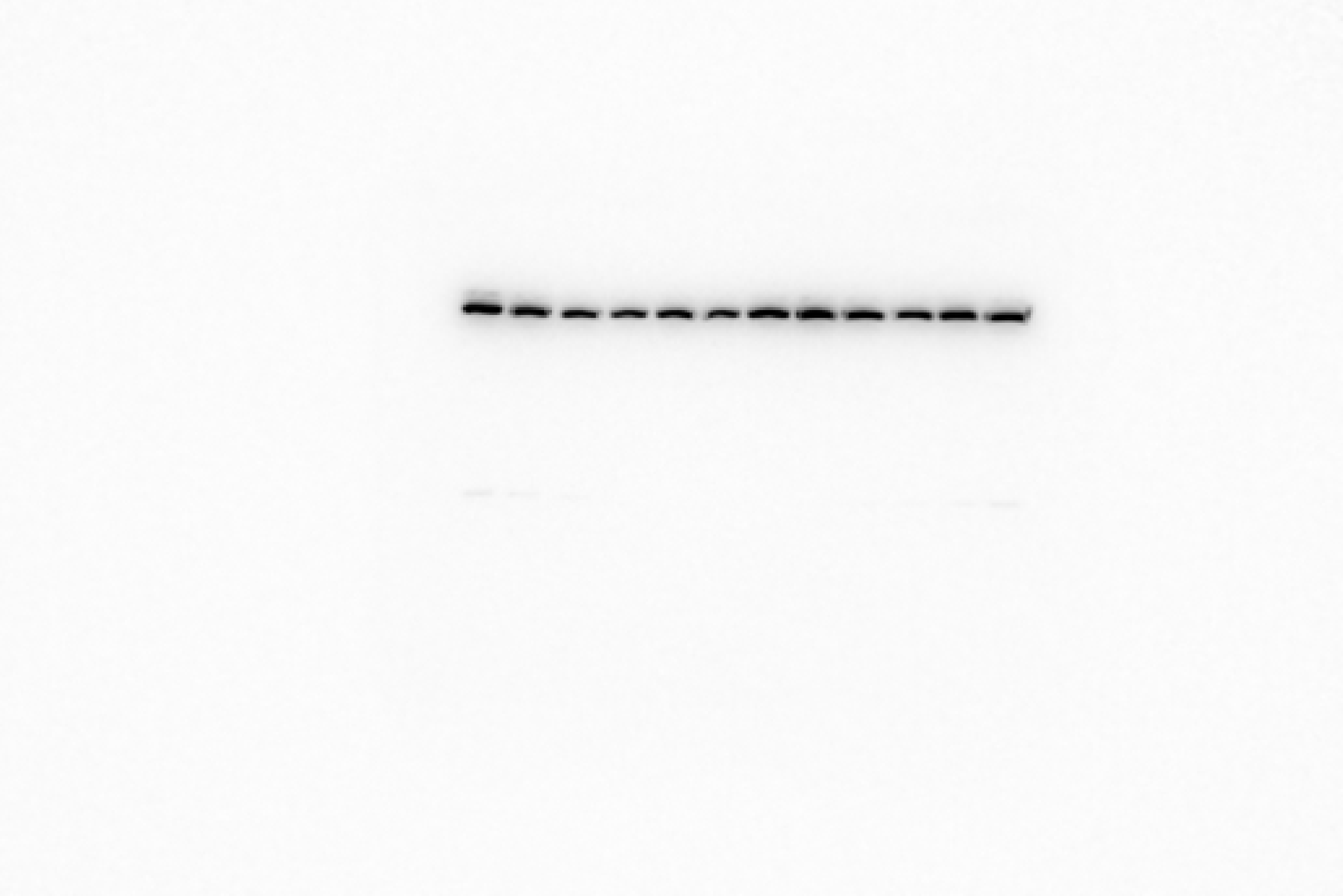

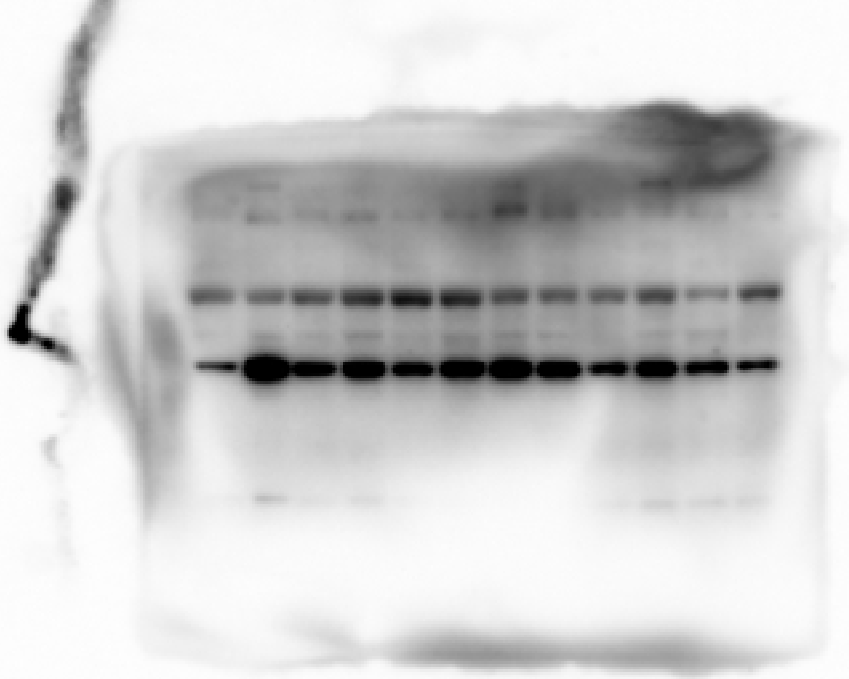

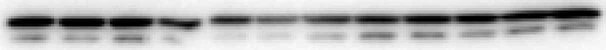

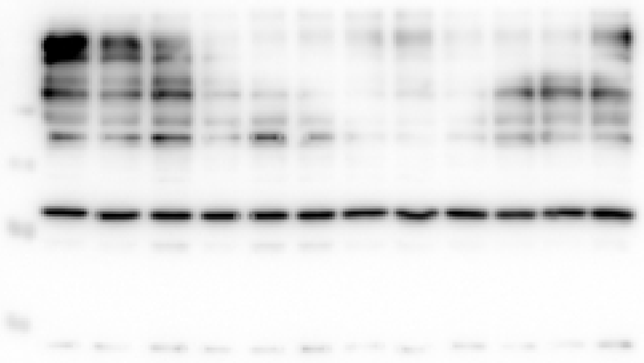

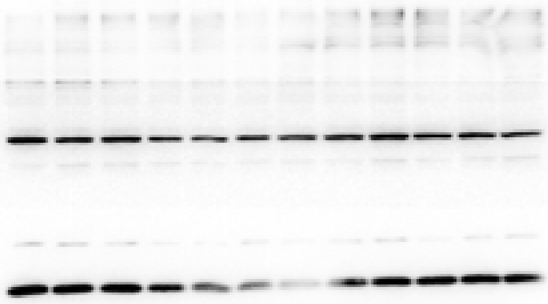

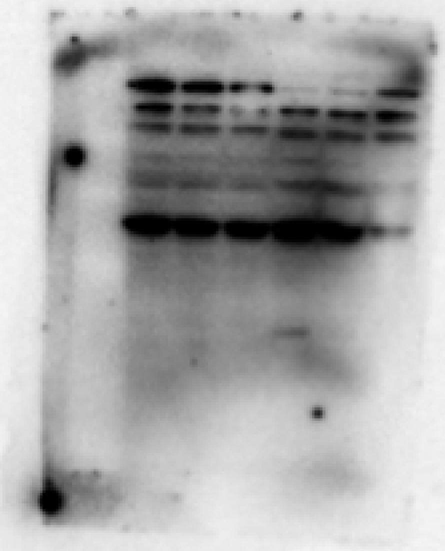

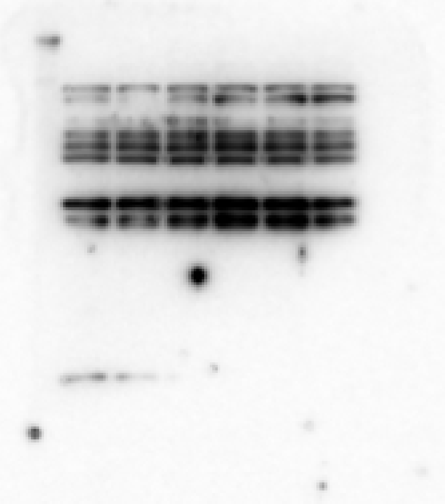

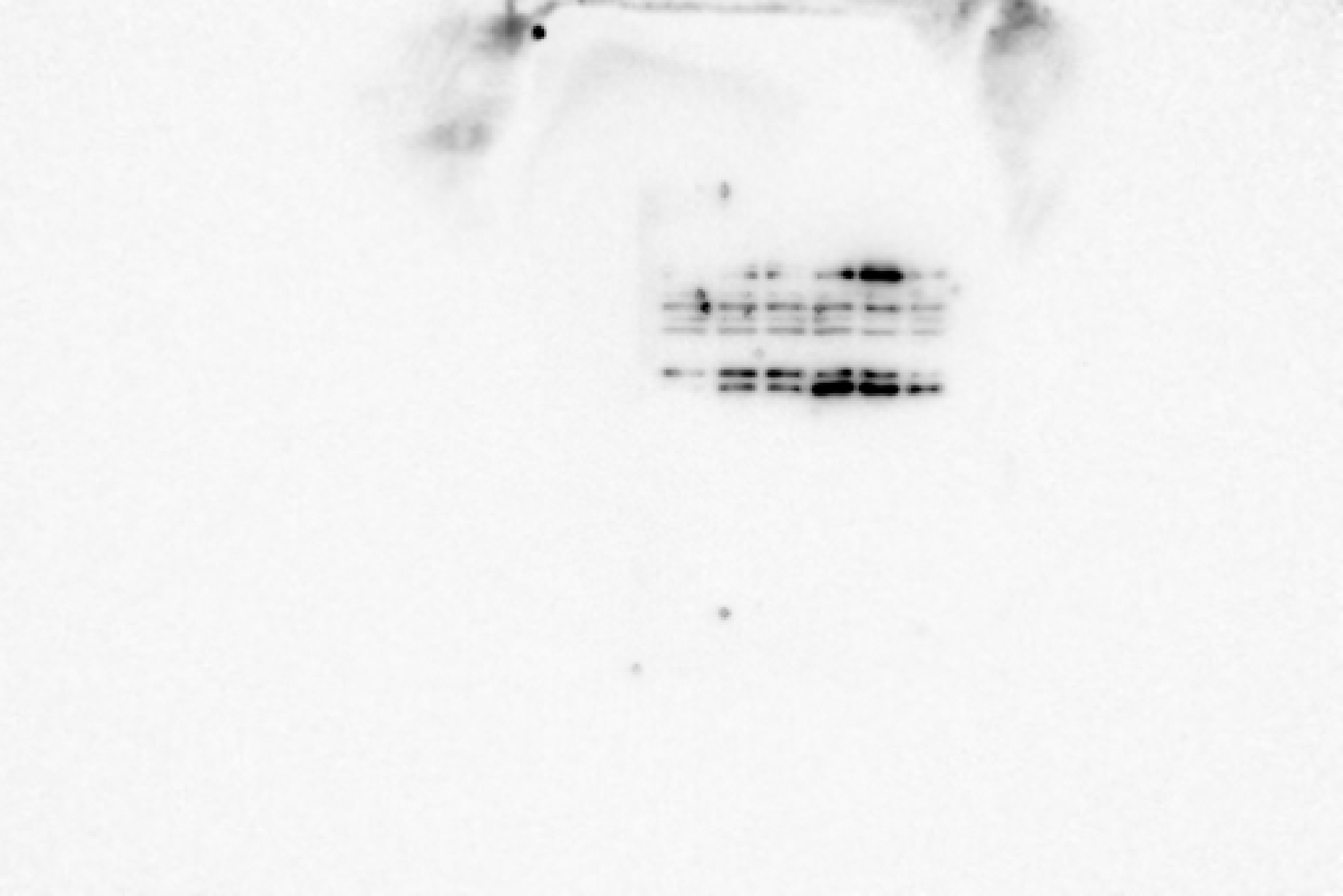

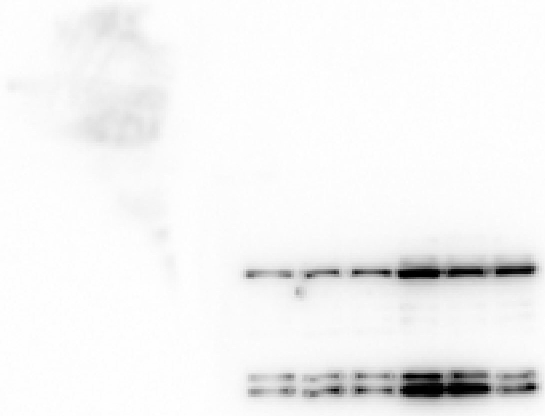

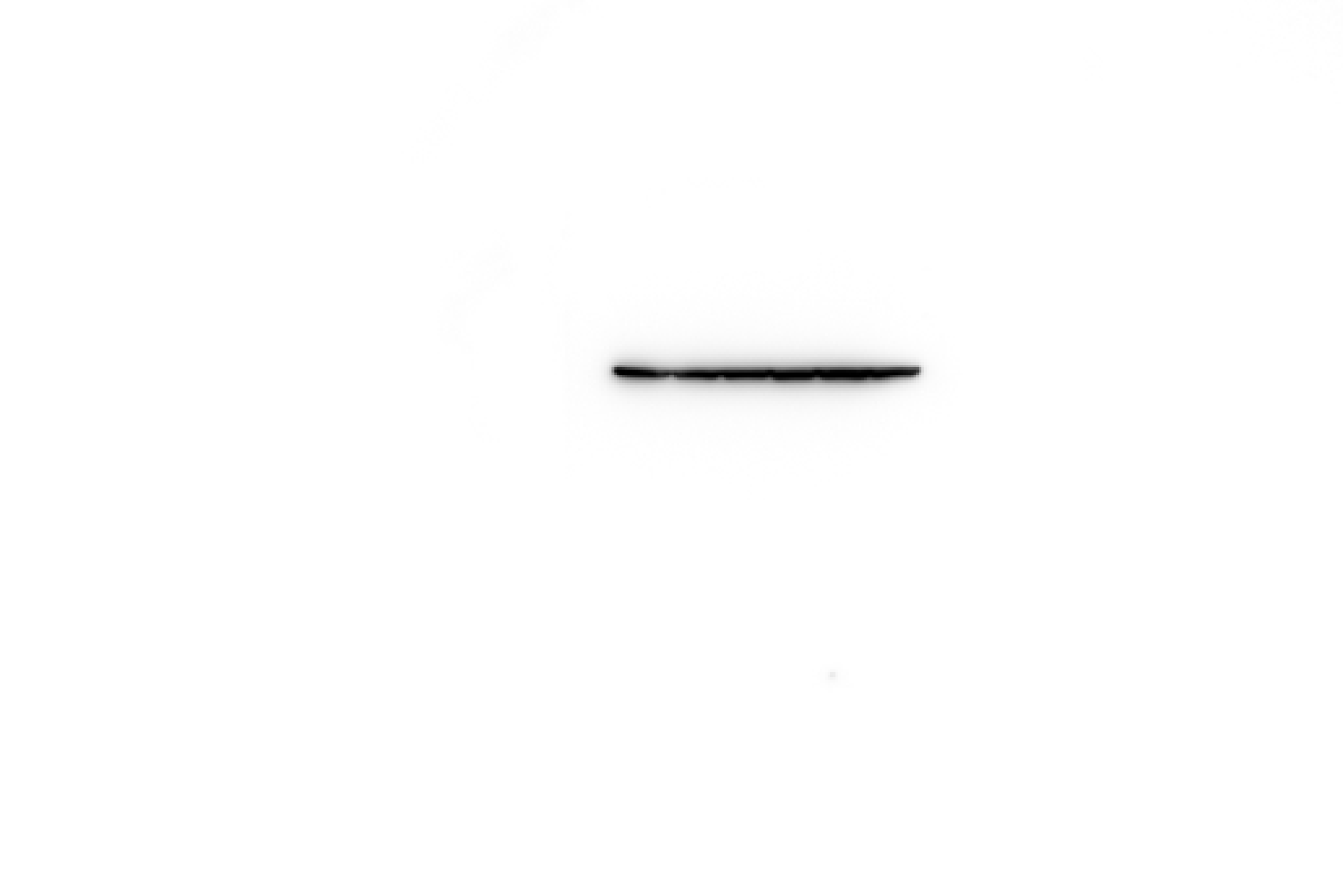

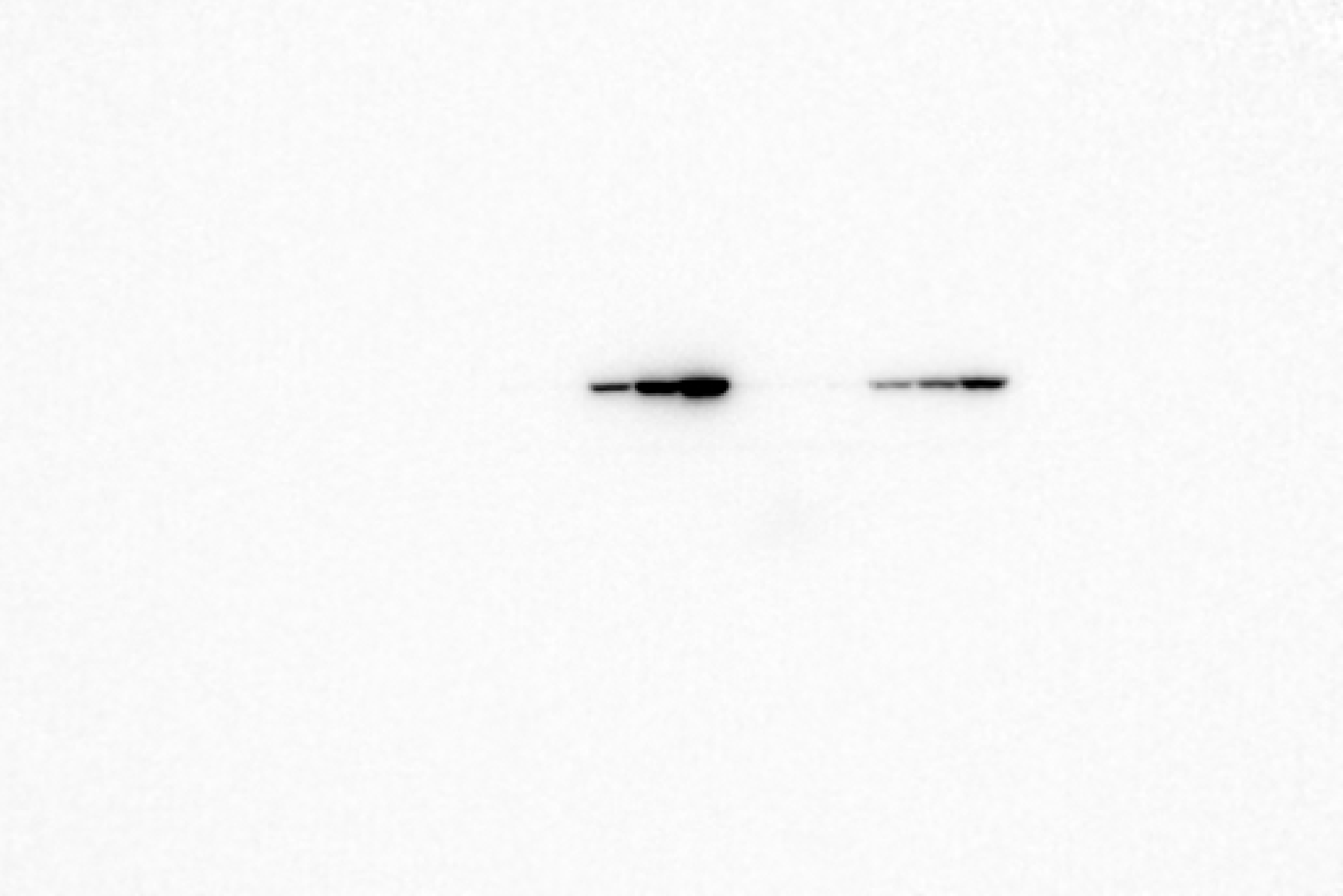

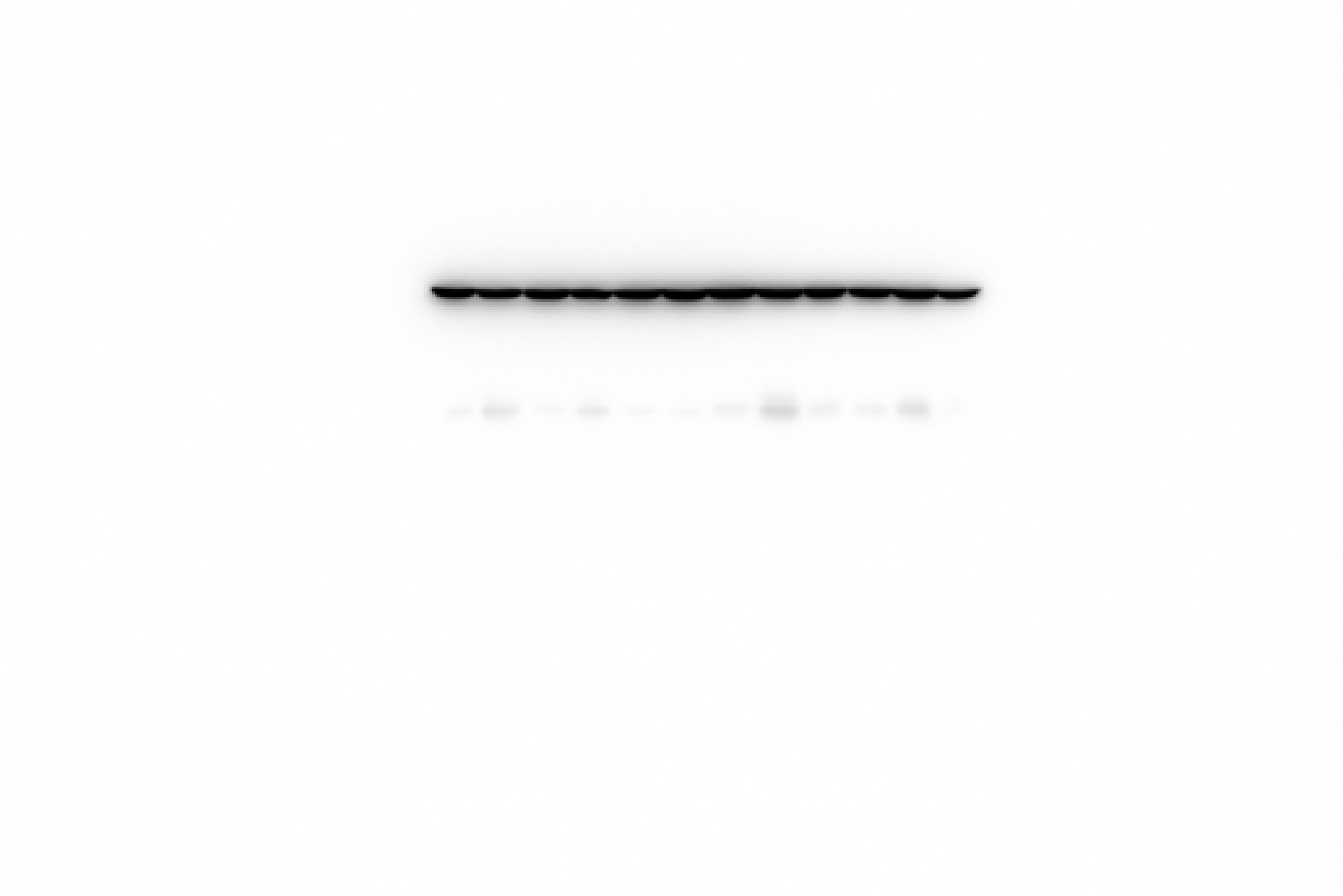

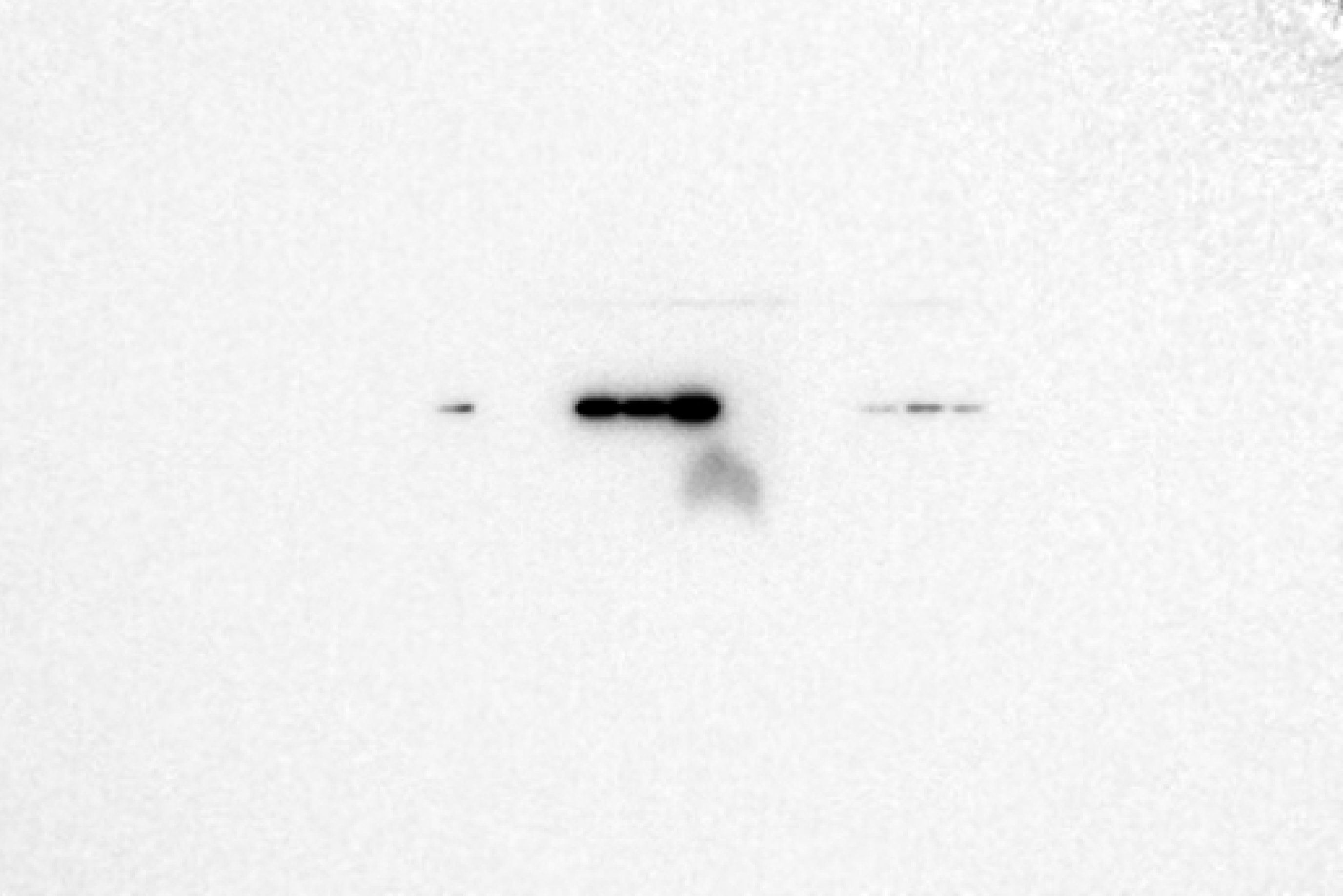

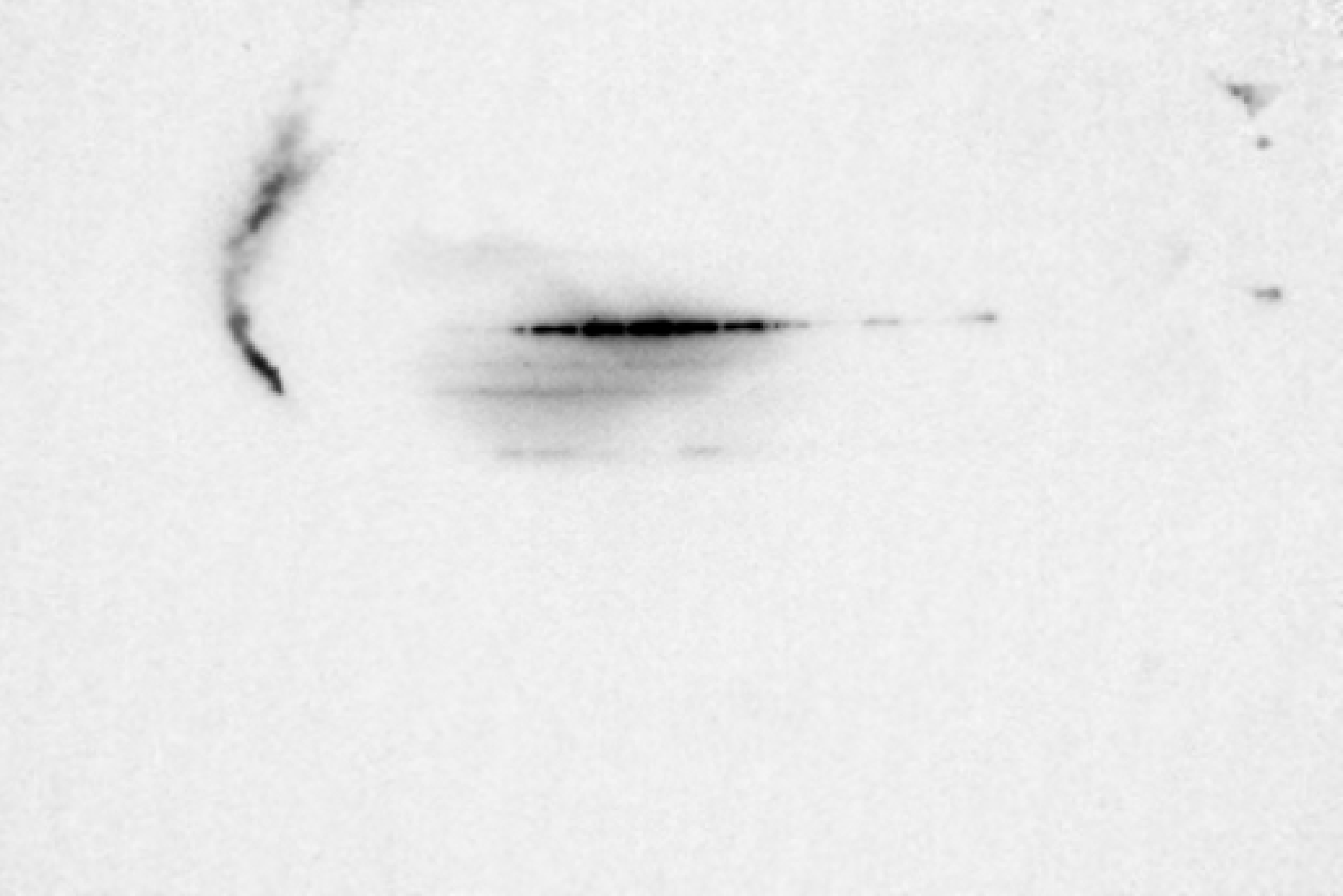

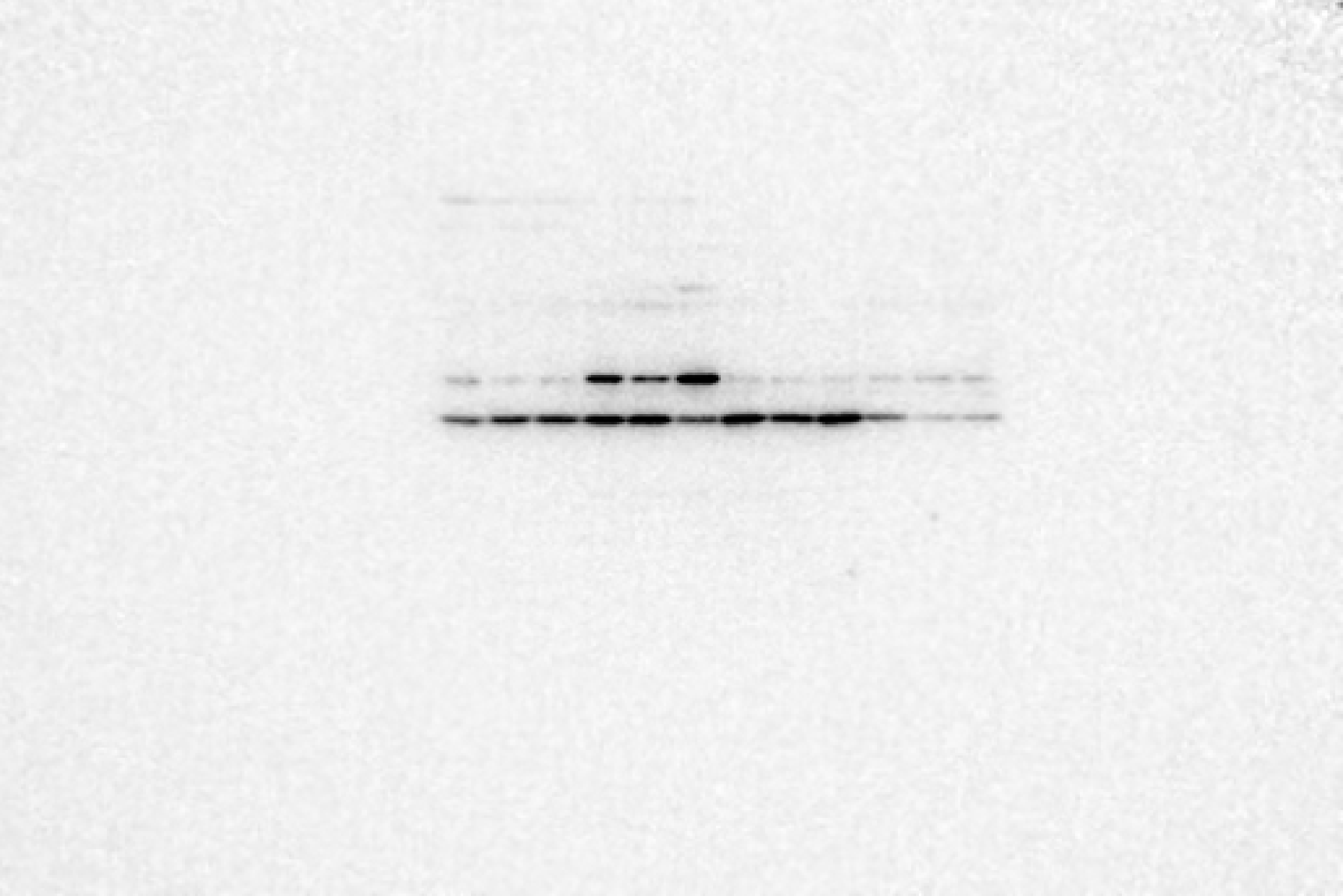

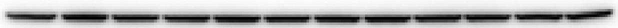

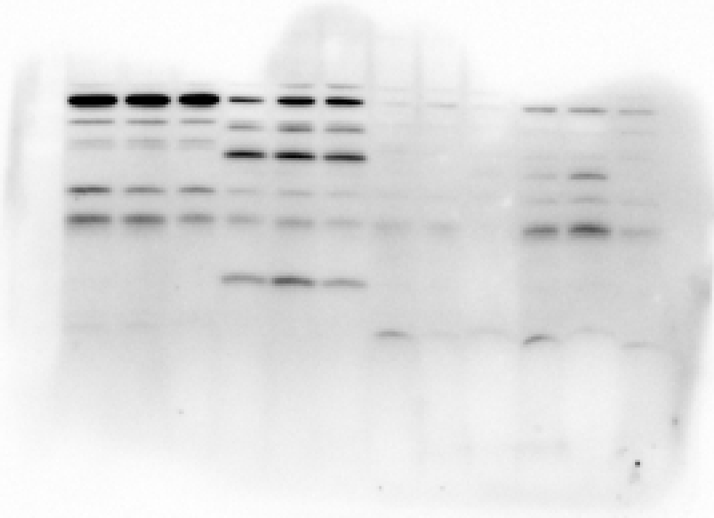

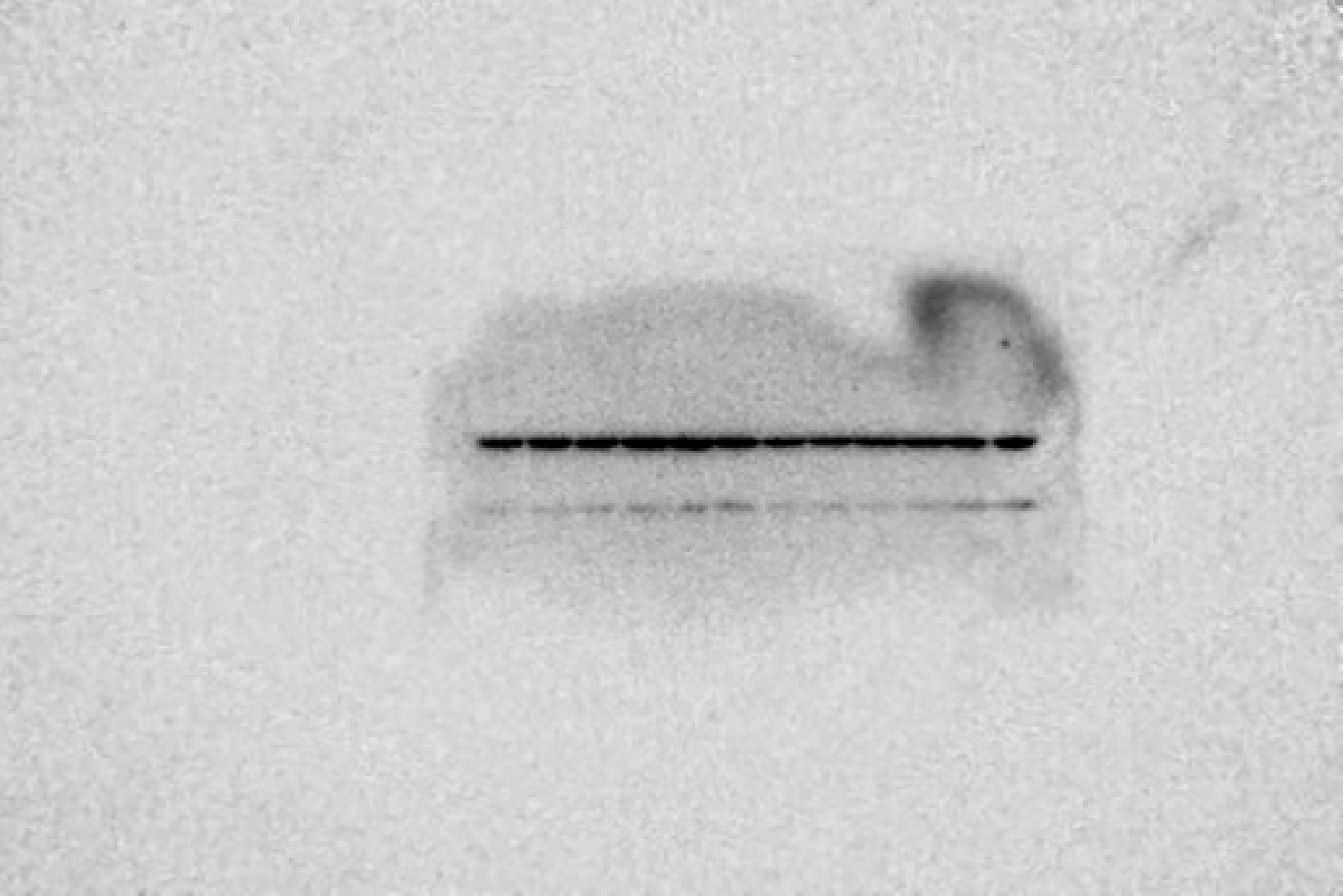

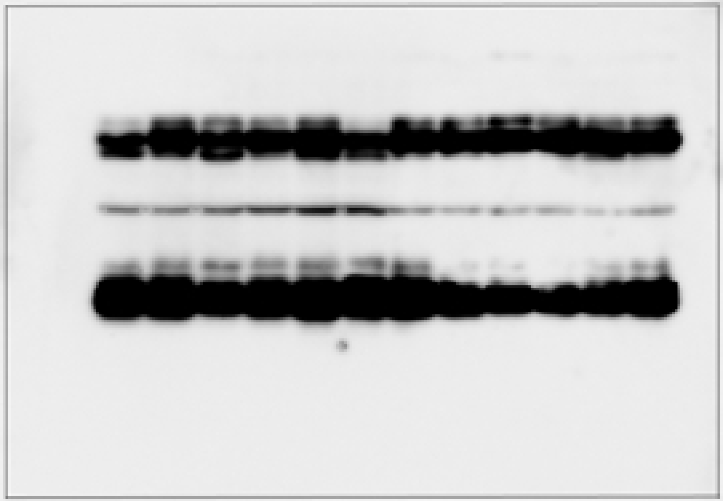

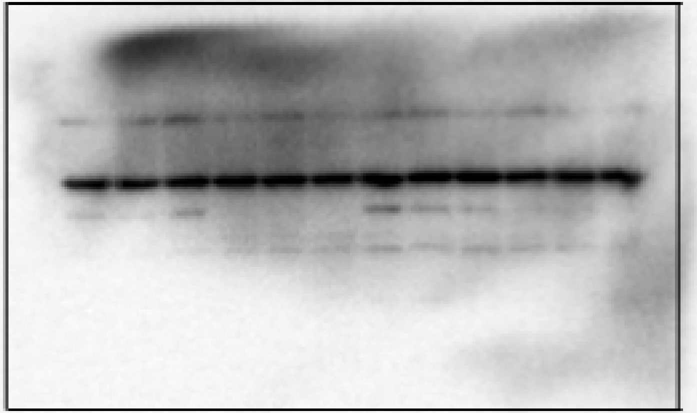

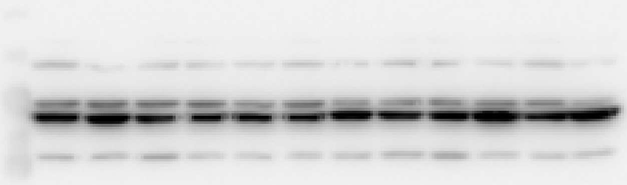

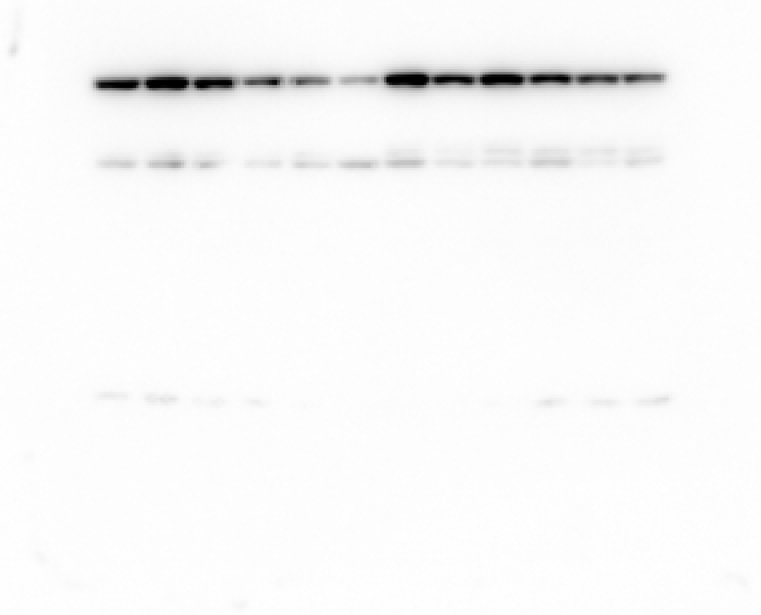

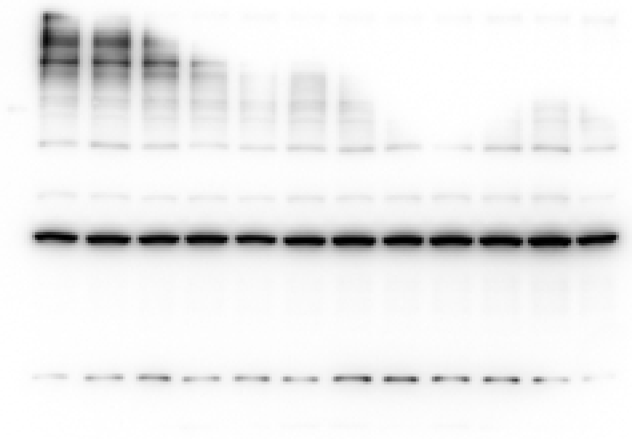

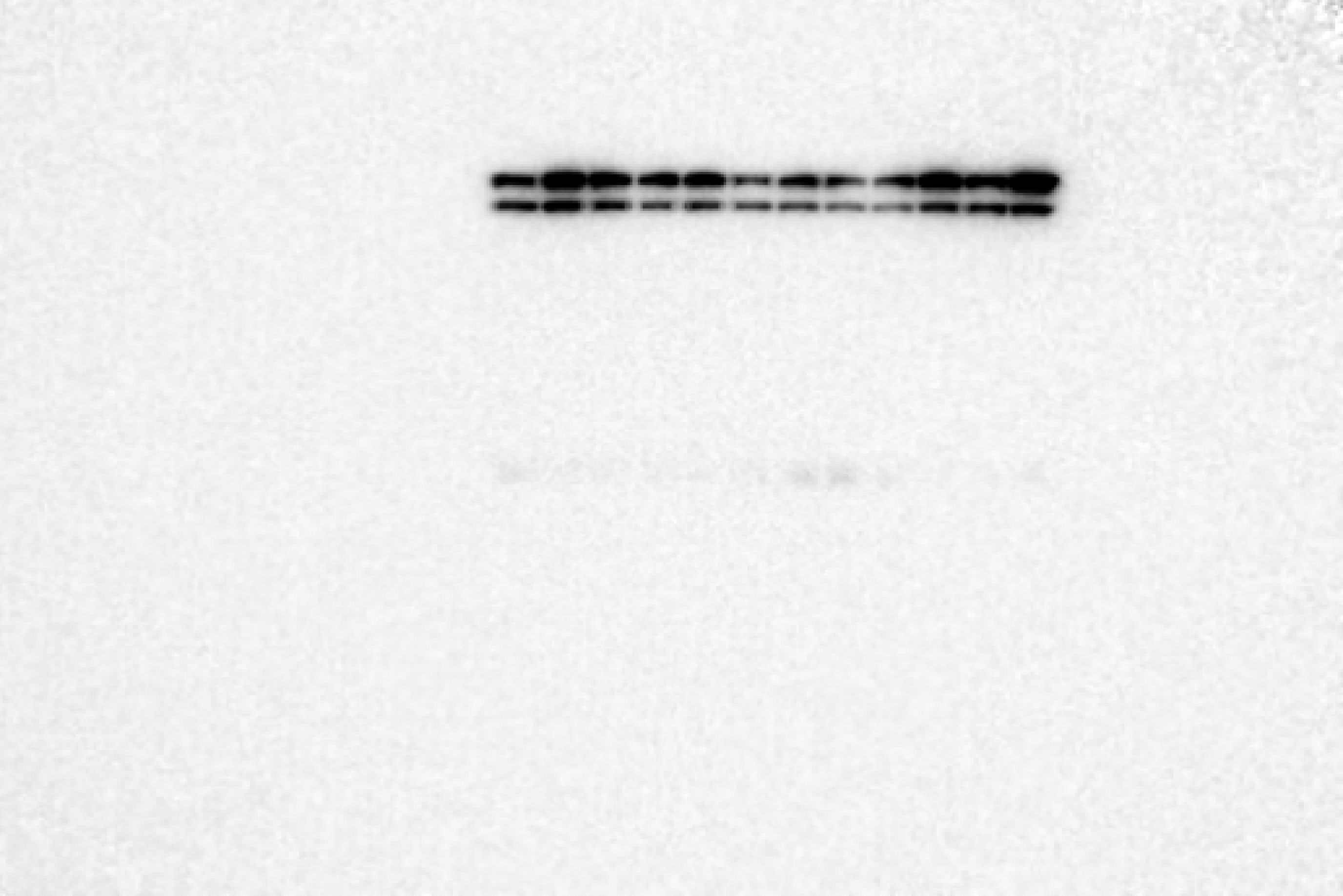

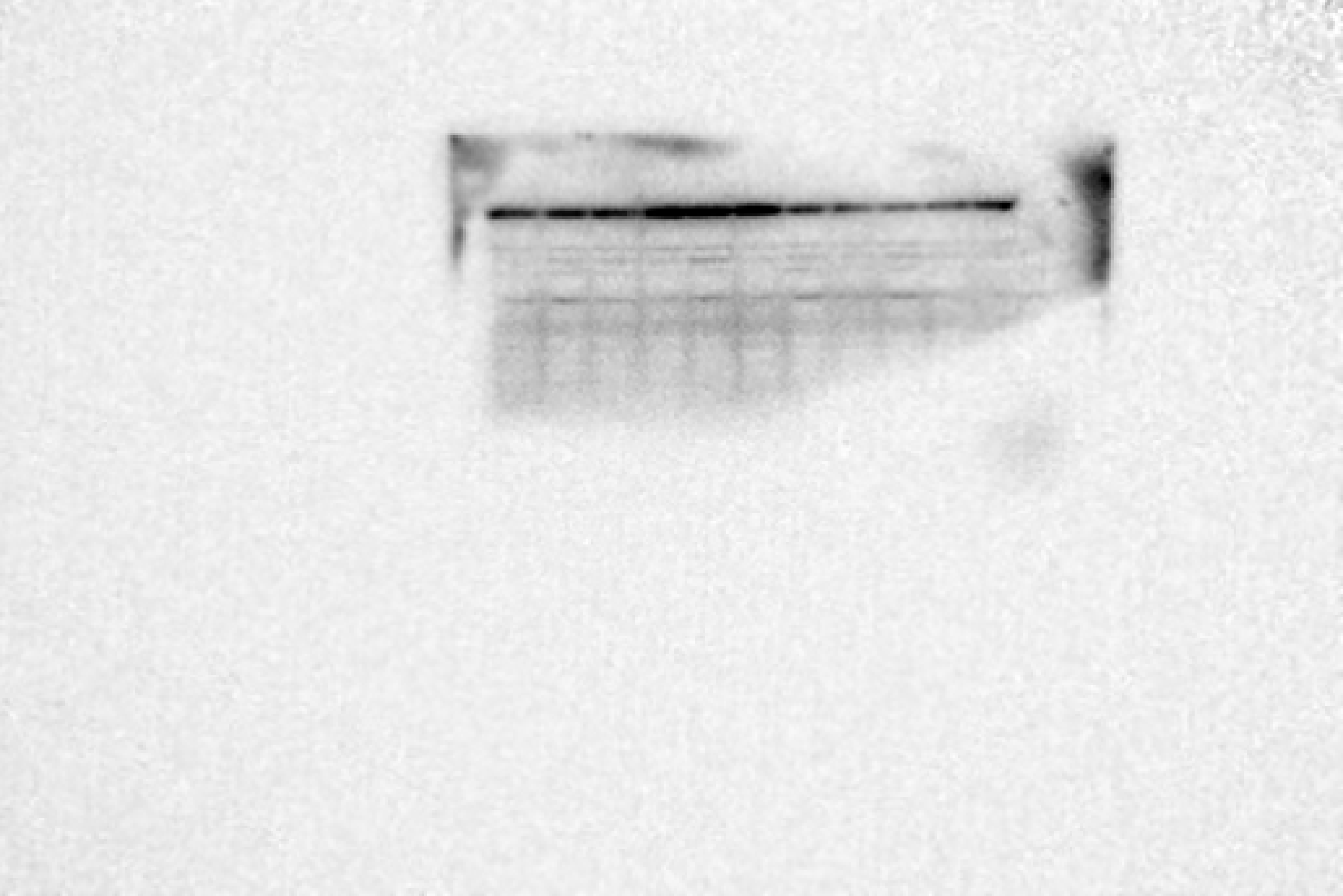

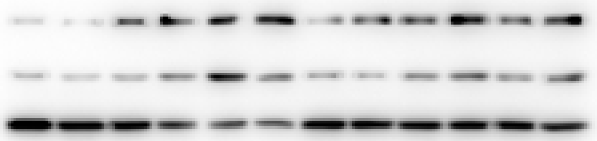

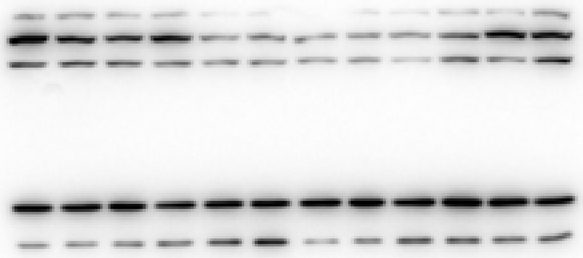

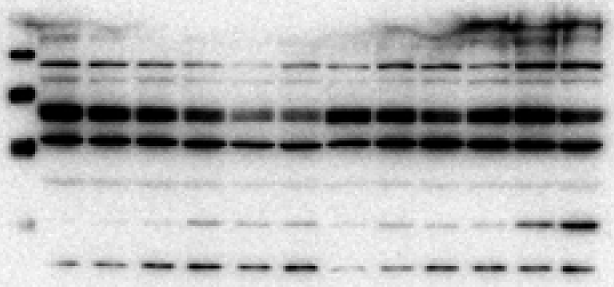

Supplement: Supplementary file 1 — Supplementary Information [file 41598_2017_2759_MOESM1_ESM.pdf]
